# Spatial variations of soil respiration and temperature sensitivity along a steep slope of the semiarid Loess Plateau

**DOI:** 10.1371/journal.pone.0195400

**Published:** 2018-04-06

**Authors:** Qiqi Sun, Rui Wang, Yaxian Hu, Lunguang Yao, Shengli Guo

**Affiliations:** 1 State Key Laboratory of Soil Erosion and Dryland Farming on the Loess Plateau, Institute of Soil and Water Conservation, Chinese Academy of Sciences and Ministry of Water Resources, Yangling, Shannxi, China; 2 University of Chinese Academy of Sciences, Beijing, China; 3 College of Resources and Environment, Northwest A&F University, Yangling, Shannxi, China; 4 Collaborative Innovation Center of Water Security for Water Source Region of Mid-line of South-to-North Diversion Project of Henan Province, Nanyang Normal University, Nanyang, China; RMIT University, AUSTRALIA

## Abstract

The spatial heterogeneity of soil respiration and its temperature sensitivity pose a great challenge to accurately estimate the carbon flux in global carbon cycling, which has primarily been researched in flatlands versus hillslope ecosystems. On an eroded slope (35°) of the semiarid Loess Plateau, soil respiration, soil moisture and soil temperature were measured *in situ* at upper and lower slope positions in triplicate from 2014 until 2016, and the soil biochemical and microbial properties were determined. The results showed that soil respiration was significantly greater (by 44.2%) at the lower slope position (2.6 μmol m^–2^ s^–1^) than at the upper slope position, as were soil moisture, carbon, nitrogen fractions and root biomass. However, the temperature sensitivity was 13.2% greater at the upper slope position than at the lower slope position (*P* < 0.05). The soil fungal community changed from being Basidiomycota-dominant at the upper slope position to being Zygomycota-dominant at the lower slope position, corresponding with increased β-D-glucosidase activity at the upper slope position than at the lower slope position. We concluded that soil respiration was enhanced by the greater soil moisture, root biomass, carbon and nitrogen contents at the lower slope position than at the upper slope position. Moreover, the increased soil respiration and decreased temperature sensitivity at the lower slope position were partially due to copiotrophs replacing oligotrophs. Such spatial variations along slopes must be properly accounted for when estimating the carbon budget and feedback of future climate change on hillslope ecosystems.

## Introduction

Soil respiration (*R*_s_) is the second-largest terrestrial carbon flux in the world [1]. Small variations in soil respiration can provoke large fluctuations in atmospheric CO_2_ concentrations [2]. Although the heterogeneity of *R*_s_ has been extensively studied across a broad range of ecosystems [3–6], there is little consensus on the spatial patterns of *R*_s_ [7], leading to considerable uncertainty when estimating soil respiration at the global scale [3]. To date, most of the studies on the spatial variability of soil respiration have been carried out on flatlands [3, 8–10]. Little is known about the spatial variation of soil respiration in hillslope ecosystems, especially considering the fact that more than 60% of the global land area is slopes with gradients >8° [11]. Therefore, it is highly relevant to characterize the spatial variation of soil respiration on sloping land, so as to accurately estimate the carbon budget in the hillslope ecosystem and improve our current understanding of erosion-induced carbon emissions. Soil respiration is, in theory, regulated both by inherent soil properties (e.g., soil water content, soil carbon content, nitrogen content and microbial communities) and climate factors (e.g., precipitation and temperature) [12–14]. However, unlike flatlands, sloping lands feature a spatial redistribution of water and soil particles via erosion [15, 16], which potentially have varying impacts on soil respiration along slopes. For instance, via overland runoff and subsurface water flows, soils at lower slope positions tend to have a greater water holding capacity [6] and potentially provide more favorable conditions for soil respiration than those at upper slope positions. Selective erosion and deposition of light/fine particles along slopes and the accompanying redistributed soil organic carbon (SOC) and other nutrients [4, 17–19] can have varying effects on crop growth, root biomass and leaf litterfall [20–22], which in turn, affect SOC accumulation and decomposition. In addition, on slopes with different gradients or orientations, solar radiation can be considerably different, resulting in distinctive temperature changes and photosynthate distribution patterns along slopes [23]. Soil microbial communities also have spatial patterns with different diversities, activities and community structures along slopes [24, 25], thereby potentially influencing the slope-scale SOC balance [26].

The temperature sensitivity of soil respiration represents the response of soil respiration to temperature changes [27]. A multitude of temperature-response functions have been used to simulate the temperature response of soil respiration [28], among which the *Q*_10_ function [3, 29] is the most widely used. The *Q*_10_ (multiplier of soil respiration rate for a 10°C increase in temperature [30]) is an important parameter when modelling the effects of global warming on terrestrial ecosystems’ carbon release [28] and, consequently, its feedback on atmospheric CO_2_ concentrations [31, 32]. The variability of *Q*_10_ (from little more than 1 to as high as more than 10) among ecosystems has been reported to mainly account for substrate quality [33] and climate factors [34], which are spatially heterogeneous. Nevertheless, *Q*_10_ may have contrasting responses with soil respiration [35, 36]. For example, the enzyme-activation theory predicted that the substrate of a recalcitrant molecular structure, which should have a less active respiration [37, 38], was degraded with a higher *Q*_10_ [39, 40]. However, there have been few systematic investigations on the spatial variations of soil respiration and *Q*_10_ on sloping land.

In this study, soil respiration, soil temperature and soil moisture were measured *in situ* at upper and lower slope positions for three years in three replicated plots of a steep-slope grassland ecosystem. Our aim was to characterize the spatial variations of soil respiration and *Q*_10_ along a steep slope and explore the potential role of soil water, root biomass, substrate availability and microbial community distribution patterns in spatial variations of soil respiration and *Q*_10_ along the steep slope. We hypothesized the following: 1) Soil respiration was greater at the lower slope position than at the upper slope position; 2) *Q*_10_ was greater at the upper slope position than at the lower slope position.

## Materials and methods

### Ethics statement

There were no specific permissions required for these locations/activities because the experimental site was located at Changwu State Key Agro-ecological Experimental Station. We confirmed that the field studies did not involve endangered or protected species.

### Study site

**Fig 1:**
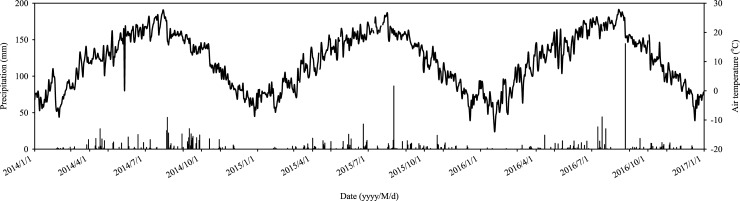
Variations of air temperature, precipitation at the experimental site from 2014 to 2016 (S1 Text).

This study was conducted on a typical ridge slope in the Wangdonggou watershed (35°13′N–35°16′N, 107°40′E–107°42′E; elevation 946–1226 m; area 8.3 km^2^), which is located in the typical eroded tableland-gully region of the southern Loess Plateau in the middle reaches of the Yellow River in northern China [41]. The soil erosion there is so rampant (soil erosion modulus of 2,860 t ha^–1^ yr^–1^) that it has greatly reduced crop yield and altered regional hydrologic regimes [42, 43]. Sloping land and gullies account for two-thirds of the watershed area. Due to the fragmented terrain, the slopes in the Loess Plateau are characterized by small slope lengths and steep slope gradients, with approximately 60% of the slopes in length < 60 m and more than 41.2% of the slopes with gradients > 25° [44]. Severe erosion areas, approximately 13% of the entire area of the Wangdonggou watershed, were mainly on steep slopes (25°–35°) [45]. Given the heavy erosion, sloping land is the main targeted area for soil erosion [46]. The region has a continental monsoon climate with a mean temperature of 9.4°C (1957–2016). The mean annual precipitation is 560 mm, 60% of which falls between July and September. Annual sunshine duration is 2,330 h, annual total radiation is 484 kJ cm^–2^, and the average frost-free period is 171 days. The meteorological data (mean daily air temperature and daily total precipitation) (Fig 1, S1 Text) were provided by the State Key Agro-Ecological Experimental Station established in 1984 in Changwu County.

According to the American Soil Classification System, the experimental soil is a uniform loam of loess deposits belonging to calcic cambisols, which originate from parent material of calcareous loess. Soil samples collected at 0–20 cm depth were characterized as follows: pH 8.3 (1:1 soil/H_2_O suspension); clay content 9.3%; silt content 44.4% and sand content 46.4%; soil bulk density 1.17 g cm^–3^; SOC 3.7 g kg^–1^; soil inorganic carbon 19.6 g kg^–1^; and stable aggregates (> 250 μm) 38.4%. Soils in this region are heavily weathered and low in SOC [47]; thus, they are highly erodible with poor cohesion.

### Experimental design

In 2014, three west-facing rectangular plots were established on a natural steep slope (35°), covered by grass. To avoid the influences of slope aspect and differences in the original soil properties on soil respiration, all three plots were established on the same slope with similar soil properties and, therefore, had the same aspects. Each plot was 20 m × 5 m with the longest side in the direction of the slope gradient. Plots were separated 150 m apart and separated by a brick wall of 15 cm in height, 40 cm in depth and 6 cm in thickness to prevent the inflow of runoff outside of the plots and the outflow of runoff to the inside of the plots. Each plot had a catchment water base, a water sink, and two water tanks (A and B) for the measurement of runoff and sediment. The water base was tilted inwardly towards the center to help water and sediment produced in the plot flow into the water sink. Two cylindrical steel buckets with an inner diameter of 80 and 90 cm and a height of 125 cm were used as the water tanks. Nine holes with the same diameter were arranged at a depth of 40 cm in water tank A. The middle hole was connected to water tank B, and the other eight holes were arranged symmetrically for the drainage of water. The 20 m length plot was divided into two parts by the 10 m boundary, above which was the range of the upper part of the plot and down which was the range of the lower part of the plot. The positions with respect to the plot are referred to as the upper slope position (upper) and lower slope position (lower), which was similar in design with that of one previous study [48]. For each plot, three polyethylene soil collars (20 cm in diameter and 12 cm in height) at each slope position, were placed at a soil depth of 10 cm in March 2014 to collect the soil respiration data *in situ*. The collars were at least 50 cm apart from each other to representatively cover the upper or lower slope position. Quadrats of 1 m × 1 m were established for the grassland to determine species (*Bothriochloa ischaemum* L.), herb coverage (75%), age (50 to 60 years old), height (0.45 m), and aboveground litter (6.1 Mg ha^–1^) [49].

### Measurements of soil respiration, soil temperature and soil moisture

The measurements were carried out from March 2014 until November 2016. During the three years of the observation periods (2014, 2015 and 2016), the respiration rates of surface soil (*R*_s_) were measured every seven days by mounting a soil CO_2_ flux system (a portable chamber of 20 cm in diameter, Li-8100, Lincoln, NE, USA) onto the polyethylene collars. When effective rainfall events occurred, measurements were conducted immediately afterwards, and continued for at least three days to collect the possible pulses of CO_2_ emissions stimulated by rainfall events [36, 41]. Given the limited soil respiration activities in the cold winters, the CO_2_ flux was only measured once a month during December, January and February. Each measurement was conducted between 9:00 am and 11:00 am [2], and all visible living organisms were removed prior to measurements. Soil respiration at each slope position was calculated from the mean of the three collar measurements (the measurement at three collars in each slope position differed by less than 15% at any measurement period).

At the same time as the soil respiration measurement, the soil temperature and soil moisture at 5 cm depth were also measured in three directions, each 10 cm away from the collar. The soil temperature was measured using a Li-Cor thermocouple probe, and the soil moisture was determined by a Theta Probe ML2X with an HH2 moisture meter (Delta-TDevices, Cambridge, England). The soil moisture was not acquired from December to February because of frost or snow cover. The soil water-filled pore space (WFPS) was calculated by the following equation [50]: WFPS (%) = [volumetric water content / 100 × (2.65 − soil bulk density) / 2.65] × 100%.

### Soil sampling and soil chemical analysis

To obtain basic soil properties, six soil samples were taken in parallel positions beside each plot (three cores at the upper slope position and three cores at the lower slope position) using a soil auger of 3 cm in diameter in the last experimental year (October, 2016). Each sample consisted of three subsamples that were randomly collected from the topsoil (0–20 cm). Immediately after sampling, each sample was passed through a 2.0 mm sieve and divided into three portions: one portion was stored at –80°C for DNA extraction, one portion was stored at 4°C for less than four days to measure soil microbial biomass carbon content (SMBC), soil dissolved organic carbon (DOC) and soil nitrate (NO_3_-N) and ammonium (NH_4_-N) nitrogen content, and the third portion was air-dried and then crushed to pass through a 0.15 mm sieve to determine the soil organic carbon (SOC). The SOC was determined using the K_2_CrO_7_-H_2_SO_4_ oxidation method [51]. The SMBC and DOC were determined by the chloroform fumigation-extraction method with a total organic carbon analyzer (TOC-VCSH, Shimadzu, Japan) [52, 53]. The portion of each soil sample without fumigation was the salt-soluble carbon (DOC) and the difference of the fuming portion minus the portion without fumigation was the SMBC. The soil nitrate (NO_3_-N) and ammonium (NH_4_-N) nitrogen were extracted with KCl (1 mol L^–1^) and determined by colorimetry using a Bran & Luebbe Ⅱ AutoAnalyser [54].

### Soil biological analysis

Three critical enzymes involved in soil carbon cycling were assayed in this study: β-D-xylosidase, β-D-glucosidase and cellobiohydrolase. The enzyme functions of β-D-xylosidase and cellobiohydrolase are to release xylose from hemicellulose and release disaccharides from cellulose [55]. The β-D-glucosidase represents enzymes associated with complex organic matter (cellulose and lignin) breakdown [56]. Fluorometric substrates linked to 4-MUB-β-D-xyloside, 4-MUB-β-D-glucoside and 4-MUB-cellobioside from Sigma (St. Louis, MO, USA) were used for assays of the hydrolytic enzyme. Enzymes assays were performed as described by a previous report [57] with slight modifications of the buffer concentrations due to the alkalinity of the soil in the present experiment. Briefly, assays were conducted by homogenizing each fresh soil sample (equivalent weight to 1.0 g dry mass soil) in 125 ml of 50 mM Tris buffer (pH 8.2) in a 200 ml screw-cap Nalgene bottle, and then stirring the mixture vigorously to maintain a uniform suspension. The soil sample, Tris buffer, 10 μM references and 200 μM fluorometric substrates were distributed into a black 96-well plate in the order as described by [57]. The plates were incubated in the dark at 25°C for 4 h until 10 μl of 0.5 M NaOH were added to stop the reaction by bringing the pH in the well to 10; the plates were read using an automatic microplate reader [57] at 365 nm excitation and 450 nm emission. The activities of soil enzymes were calculated using standard equations on a per gram dry soil basis [58, 59] and expressed in units of nmol g^−1^ h^−1^. To minimize root heterogeneity, six soil cores (0–20 cm) were taken in parallel positions beside each plot (three cores at upper slope position and three cores at the lower slope position) using a sharp iron tube (9 cm in diameter) and mixed well for the measurement of fine root biomass (< 2 mm). Roots were separated from soils by soaking in water and gently washing through a 0.25 mm mesh. Wet roots were then oven-dried at 60°C for 48 h to a constant weight.

### Illumina HiSeq high-throughput sequencing

Total genome DNA from the sample was extracted using the CTAB/SDS method. The DNA concentration and purity were monitored on 1% agarose gels. According to the concentration, the DNA was diluted to 1 ng/μl using sterile water. The 515F (5'-GTGCCAGCMGCCGCGGTAA-3') and 806R (5'-GGACTACHVGGGTWTCTAAT-3') were designed to amplify the hypervariable V4 region of the 16S rRNA gene from the bacteria; and the fungal ITS1 genes were amplified using primers 1737F (5'-TCCGTAGGTGAACCTGCGG-3') and 2043R (5'-GCTGCGTTCTTCATCGATGC-3') [60]. We mixed the same volume of 1X loading buffer (constrained SYB green) with PCR products and operated electrophoresis on a 2% agarose gel for detection. Samples with a bright main strip between 400–450 bp were chosen for further experiments. The PCR products were mixed in equidensity ratios. Then, the mixture of the PCR products was purified with Qiagen Gel Extraction Kit (Qiagen, Germany). Sequencing libraries were generated using TruSeq® DNA PCR-Free Sample Preparation Kit (Illumina, USA) following the manufacturer’s recommendations and index codes were added. The library quality was assessed in the Qubit® 2.0 Fluorometer (Thermo Scientific) and Agilent Bioanalyzer 2100 system. Sequencing was conducted on an Illumina HiSeq 2500 platform (Illumina Corporation, San Diego, USA) and 250 bp paired-end reads were generated. Approximately 80,208 high-quality prokaryotic sequences per sample with an average length of approximately 253 bp, and 66,221 high-quality eukaryotic sequences per sample with an average length of approximately 239 bp were produced. A sequence analysis was performed by the UPARSE software package using the UPARSE-OTU and UPARSE-OTUref algorithms [61]. Alpha-diversity indices including the Chao1 estimator of richness, abundance-based coverage estimator (ACE) and Shannon’s diversity index were generated based on the obtained Operational Taxonomic Units (OTUs). The high-throughput sequencing data are available in the NCBI Sequence Read Archive (SRA) database (Accession numbers SUB2982447 and SUB2559665).

### Data analysis

An exponential function was used to represent the relationship between the soil respiration rate and soil temperature [1]:

$$y=\beta_{0}e^{\beta_{1}T}$$ (1)

where *y* (μmol m^–2^ s^–1^) is the measured soil respiration rate, T (°C) is the measured soil temperature at 5 cm depth, and *β*_0_ and *β*_1_ are constants fitted by the least squares method.

The temperature sensitivity of soil respiration (*Q*_10_) was calculated by Eq (2) [3]:

$$Q_{10}=e^{10\beta_{1}}$$ (2)

Soil respiration, soil temperature, soil moisture, soil biochemical properties and microbial properties were subject to the two independent samples T-test to detect the difference between slope positions. The statistical significance was defined as *P* ≤ 0.05. All of the statistical analysis was performed using SPSS 20.0 software (SPSS Inc., Chicago, USA). The figures were generated using Sigmaplot 12.5 software (Systat Software Inc., San Jose, CA, USA) (http://dx.doi.org/10.17504/protocols.io.nrzdd76).

## Results

### Soil respiration and *Q*_10_ at upper and lower slope positions

**Fig 2:**
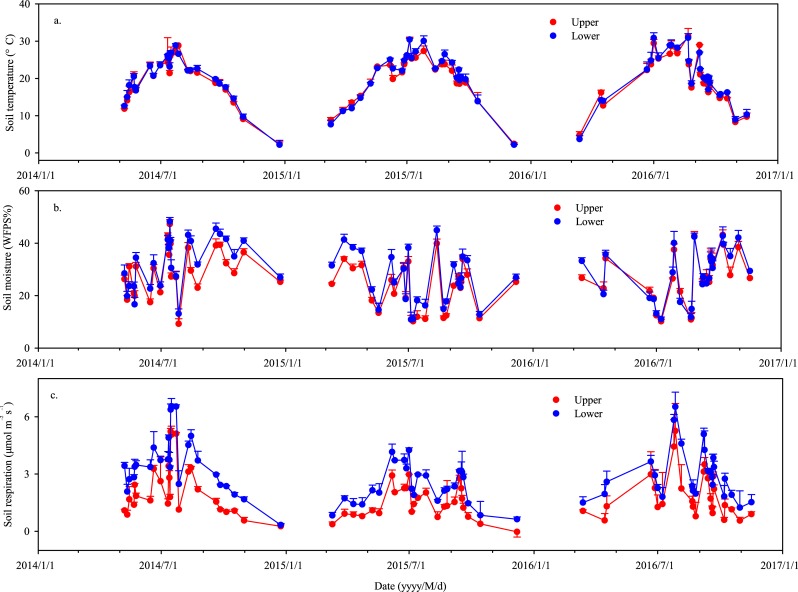
Dynamics of soil temperature (a.), soil moisture (b.) and soil respiration rate (c.) at upper and lower slope positions over the study years (±SE, n = 3) (S2 Text).

The soil temperature at the upper and lower positions had similar seasonal and annual patterns over the period of three years (Fig 2A, S2 Text), which was in good agreement with the variation of air temperature (Fig 1, S1 Text). However, the mean soil temperature varied little between slope positions (24.9°C and 25.2°C for upper and lower slope positions, respectively, *P* > 0.05) (Table 1). The soil moisture (%WFPS) (Fig 2B, S2 Text), in general, corresponded well with precipitation patterns (Fig 1, S1 Text) and was 15% greater at the lower slope position (29.0% on average) than at the upper slope position (25.2% on average, *P* < 0.05) (Table 1).

**Table 1:** Mean soil temperature, soil moisture, soil respiration rate (*R*_s_) and cumulative soil respiration (CO_2_-C) at upper and lower slope positions in 2014, 2015 and 2016.

Years	Slope positions	Soil temperature/ °C	Soil moisture/ %WFPS	*R*_s_/ μmol m^–2^ s^–1^	CO_2_-C/ g C m^–2^ yr^–1^
2014 (*n* = 25)	Upper	24.9±1.8 a	26.6±1.6 a	1.90±0.25 a	574.6±49.0 a
	Lower	25.3±1.2 a	29.3±2.1 b	2.59±0.32 b	796.7±33.3 b
2015 (*n* = 24)	Upper	24.3±1.1 a	23.2±1.6 a	1.26±0.12 a	350.8±60.5 a
	Lower	24.6±1.2 a	28.8±1.6 b	2.09±0.18 b	589.9±69.3 b
2016 (*n* = 26)	Upper	25.5±1.4 a	25.7±1.8 a	2.27±0.46 a	658.5±69.6 a
	Lower	25.8±1.6 a	28.8±1.9 b	3.14±0.37 b	913.1±74.8 b
Average (*n* = 75)	Upper	24.9±1.4 a	25.2±1.7 a	1.81±0.33 a	528.0±91.8 a
	Lower	25.2±1.4 a	29.0±1.9 b	2.61±0.32 b	766.6±94.5 b

Note: Different letters indicate significant difference at *P* < 0.05, and values are means of three replicates ± SE.

Soil respiration rates (*R*_s_) agreed well with the soil temperature changes (Fig 2A, S2 Text), and showed similar seasonal and annual patterns at the upper and lower slope positions (Fig 2C, S2 Text). Within each season, the soil respiration rates also fluctuated in response to the soil moisture, rising gradually from March to June, peaking in the rainy season and declining fast after October (Fig 2C, S2 Text). Furthermore, the average *R*_s_ at the lower slope position were 36.2%, 65.8% and 38.2% greater (*P* < 0.05) than those at the upper slope positions in 2014, 2015 and 2016, respectively (Table 1). After averaging the three years, the mean soil respiration was 44.2% greater at the lower slope position than that at the upper slope position (2.61 *vs*. 1.81 μmol m^–2^ s^–1^, respectively, *P* < 0.05). The cumulative soil respiration (CO_2_-C) was 48.5% greater at the lower slope position than at the upper slope position (766.6 *vs*. 528.0 g C m^–2^ yr^–1^, respectively, *P* < 0.05). In addition, the differences of *R*_s_ between the upper and lower slope positions were more pronounced during the rainy season (Fig 2C, S2 Text), where abundant precipitation was synchronized with warm temperatures (Fig 1, S1 Text). Furthermore, the exponential regression analysis indicated that the spatial variance between the slope positions not only significantly affected the soil respiration but also altered its temperature sensitivity (*Q*_10_). With the three years’ data set, the derived *Q*_10_ values for each collar followed the order of the upper slope position > the lower slope position (Table 2). After averaging the three collars, the mean *Q*_10_ was on average 13.2% greater at the upper slope position than at the lower slope position over the slope (1.95±0.07 *vs*. 1.72±0.01, *P* < 0.05).

**Table 2:** The *Q*_10_ and the relationship between soil respiration (F) and soil temperature (T) and at upper and lower slope positions for 2014, 2015 and 2016.

Years	Slope positions	Exponential equations	*N*	*r*^2^	*P*	*Q*_10_
2014	Upper	F = 0.334*e*^0.0604T^	25	0.55	<0.01	1.83
	Lower	F = 0.630*e*^0.0552T^	25	0.69	<0.01	1.74
2015	Upper	F = 0.353*e*^0.0660T^	24	0.81	<0.01	1.93
	Lower	F = 0.635*e*^0.0530T^	24	0.90	<0.01	1.70
2016	Upper	F = 0.269*e*^0.0728T^	26	0.67	<0.01	2.07
	Lower	F = 0.633*e*^0.0543T^	26	0.87	<0.01	1.72

Note: N is the number of statistical variables; *r*^2^ is the determinant coefficient; *P* is the significance level.

### Soil biochemical properties at upper and lower slope positions

The soil carbon content tended to be enriched in soils at the lower slope position when compared to that at the upper slope position, where the relative increases of SOC, DOC and SMBC were 66.7%, 74.7% and 83.1%, respectively (Table 3, *P* < 0.05). Similarly, soil mineral N significantly increased at the lower *vs*. upper slope positions (by 27.2%, *P* < 0.05). It was notable that the DOC/SOC was significantly greater at the lower slope position than that at the upper slope position, whereas SMBC/SOC was numerically but non-significantly different between the two slope positions.

**Table 3:** Soil biochemical properties at upper and lower slope positions.

Items	Upper	Lower	Increase/%
SOC/g kg^–1^	7.26±0.10 a	12.1±0.15 b	66.7
DOC/mg kg^–1^	57.40±1.10 a	100.3±7.60 b	74.7
SMBC/mg kg^–1^	146.30±13.80 a	267.8±46.30 b	83.1
(DOC/SOC)/%	0.79	1.24	56.6
(SMBC/SOC)/%	2.02	2.21	9.8
Soil mineral N/mg kg^–1^	3.24±0.12 a	4.13±0.12 b	27.2
root biomass/t ha^–1^	1.09±0.02 a	1.88±0.04 b	72.5
β-D-xylosidase/nmol g^–1^ h^–1^	3.55±0.42 a	4.96±0.58 b	28.4
β-D-glucosidase/nmol g^–1^ h^–1^	28.40±2.45 b	15.20±1.32 a	–46.5
cellobiohydrolase/nmol g^–1^ h^–1^	1.30±0.38 a	1.96±0.03 b	33.7

Note: SOC is the total soil organic carbon; DOC is the soil dissolved organic carbon; SMBC is the soil microbial biomass carbon; DOC/SOC is the proportion of dissolved organic carbon in total soil organic carbon; SMBC/SOC is the proportion of soil microbial biomass carbon in total soil organic carbon. Soil mineral N is the sum of nitrate and ammonium nitrogen. Different letters indicate the significant difference at *P* < 0.05, and values are the means of three replicates ± SE (*n* = 3).

A significant between-position difference was also observed in the root biomass, with an increase of 72.5% at the lower than at the upper slope position (1.88 *vs*. 1.09 kg m^–2^, respectively; Table 3). Among the enzyme activities, the β-D-glucosidase activities were 42.5% greater at the upper slope position than at the lower slope position (Table 3); in contrast, those of β-D-xylosidase and cellobiohydrolase were 28.4% and 33.7% greater, respectively, at the lower slope position than at the upper slope position.

### Soil microbial properties at upper and lower slope positions

A total of 721,871 and 595,992 high-quality sequences per sample were obtained for the bacterial 16S gene and fungal ITS gene from all soil samples, of which a total of 8,603 and 2,038 OTUs were identified for soil bacterial and fungal communities, respectively. The alpha diversity indices of the bacterial 16S rRNA gene were significantly greater at the lower slope position than at the upper slope position(*P* < 0.05). However, the alpha diversity indices of the fungal ITS gene were numerically but non-significantly different between the two slope positions (*P* > 0.05) (Table 4).

**Table 4:** Soil bacterial and fungal diversity indices at 97% sequence similarity of 16S rRNA and ITS gene sequence calculated based on 80,208 and 66,221 sequences for each sample.

Alpha-diversity indices		Upper	Lower	Increase/%
OTU number	Bacterial 16S	4385±58 a	4784±94 b	9.1
	Fungal ITS	662±29 a	867±78 a	31.0
ACE estimator of richness	Bacterial 16S	4416.9±73.8 a	4871.9±93.7 b	10.3
	Fungal ITS	700.1±36.4 a	928.5±102.9 a	32.6
Chao1 estimator of richness	Bacterial 16S	4318.7±71.7 a	4664.5±75.5 b	8.0
	Fungal ITS	684.0±25.5 a	663.6±175.8 a	3.0
Shannon diversity index	Bacterial 16S	9.64±0.07 a	10.01±0.04 b	3.8
	Fungal ITS	4.76±0.43 a	5.61±0.24 a	17.8

Note: Values with different letters in a column mean significant difference at *P* < 0.05, values are means of three replicates ± SE (*n* = 3).

**Fig 3:**
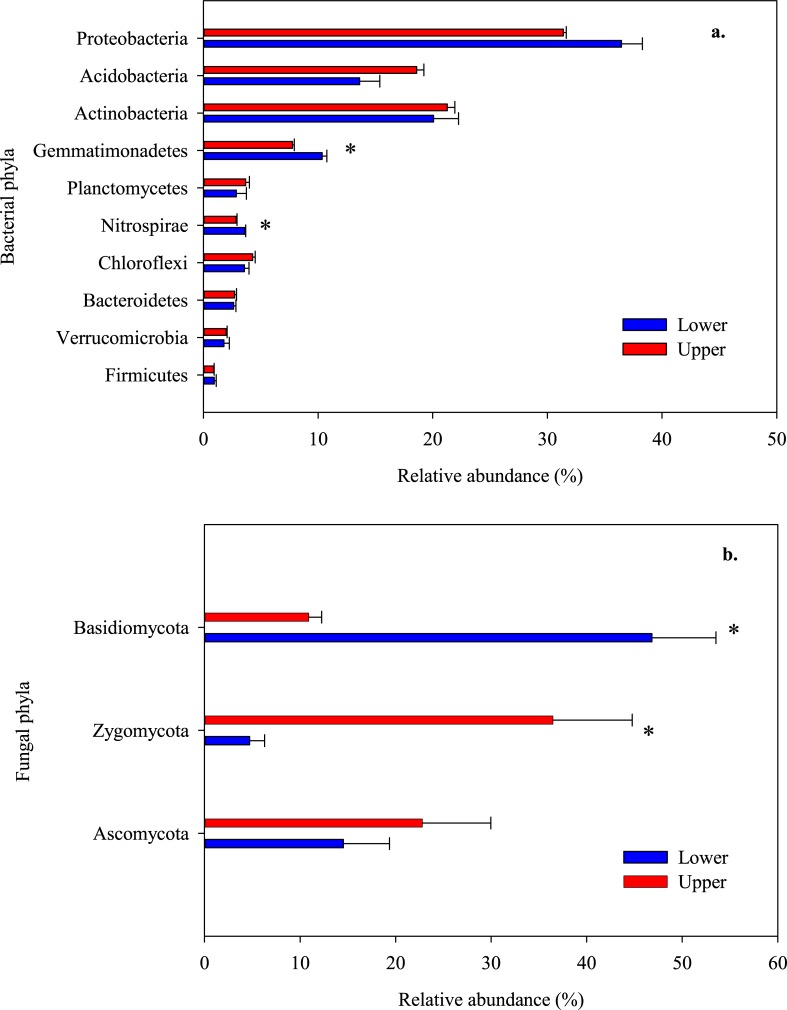
Relative abundances of the dominant bacterial (a.) and fungal phyla (b.) at upper and lower slope positions. Relative abundances are based on the proportional frequencies of the DNA sequences that could be classified (S3 Text). *indicates that the effect between upper and lower slope positions is significant.

The dominant bacterial phyla were Proteobacteria (35.1% of the total bacterial sequences), Actinobacteria (15.1%), Acidobacteria (20.9%), Gemmatimonadetes (9.5%), Planctomycetes (3.0%), Nitrospirae (3.7%), Chloroflexi (3.7%), Bacteroidetes (2.8%), Verrucomicrobia (1.8%) and Firmicutes (0.9%). Most of them had comparable relative abundances at the two slope positions except for Gemmatimonadetes and Nitrospirae, both of which were significantly greater at the upper slope position than at the lower slope position (Fig 3A, S3 Text, *P* < 0.05). The dominant fungal phylum were Basidiomycota (34.4% of the total fungal sequences), Zygomycota (17.5%) and Ascomycota (17.5%). The relative abundance of Zygomycota was significantly greater at the lower slope position than at the upper slope position (by 7.7 times, *P* < 0.05) and that of Basidiomycota showed the opposite trend (the upper slope position was 4.3 times the lower slope position, *P* < 0.05). The relative abundances of phylum Ascomycota between slope positions were numerically but not significantly different (Fig 3B, S3 Text).

## Discussion

### Spatial variation of soil respiration along the steep slope

The significantly greater soil respiration at the lower slope position compared with the upper slope position (by 44.2%, Table 1) clearly illustrated the effect of slope positions on the spatial distribution of soil respiration, which provides the evidence that supports the first hypothesis. Due to the dramatically increased fine root biomass (Table 3), this variation was largely attributable to the response of root respiration and less to that of microbial respiration. First, the greater soil respiration at the lower slope position than at the upper slope position was considerably stimulated by its more favorable soil water condition (Table 1), as soil moisture was one of the most relevant factors regulating soil respiration in the arid and semiarid study area [3, 21]. Topography, via influencing the water spatial distribution, affects the relationships between slope position and soil respiration [6]. To be specific, after receiving a comparable amount of precipitation, erosion-induced overland flow [62] and gravity-driven water flow in the subsoil [63] jointly redistributed the water along the slope. During rainfall events, the upper slope positions generally receive less inflow and move out more outflow downwards, and *vice versa* for the lower slope positions [48]. This, in sequence, accumulates water at the lower slope position with a soil moisture content of 29.0% (compared to the 25.5% at the upper slope position, *P* < 0.05) (Table 1). It is possible that more available soil water stimulated the soil respiration by enabling substrate availability [21], or by promoting root activity (i.e., root biomass in this study, Table 3) and photosynthetic rate [13], or through enhancing microbial activity and facilitating the access of the soil microbial communities to the substrates [43]. Similar patterns were observed in a semiarid grassland where a 6.0% greater soil respiration was accompanied with 1.4% higher soil moisture in the lower than upper slope positions [21]. However, the soil CO_2_ emission was observed to be independent of the slope of an evergreen broad-leaved forest ecosystem despite the significantly changed soil water content among the slope positions [64]. This indicates that soil respiration is influenced by other factors in addition to soil water, such as the amount of organic materials.

Apart from the potential effect of soil moisture, the greater contents of soil C and N fractions (Table 3) also accounted for the prevailing soil respiration observed at the lower slope position. Water erosion disturbed the carbon-rich topsoil and preferentially removed the finer particles and associated SOC from the upper slope position to the lower slope position [65, 66], which ultimately led to the significantly greater SOC content at the lower slope (Table 3). In particular, both the contents and proportions of the labile organic matter (SMBC, DOC, soil mineral N and DOC/SOC, Table 3) were significantly greater at the lower slope position than at the upper slope position. The greater labile organic matter mainly originated from the selective erosion of light and enriched particles off the upper slope position [67], or from enhanced plant C inputs to soils (such as photosynthesis and root residues) by soil water availability [13], providing an abundant substrate for microbial organisms to have more active respiration [37, 38]. Greater available C and N availability can increase microbial and root metabolic activity directly [68, 69] or indirectly, via promoted photosynthesis rates [13], soil microbial population size and microbial activity [41]. Such patterns are in line with a previous study [64], which reported more prominent soil respiration in soils with high C contents in forest ecosystems.

### Spatial variation of *Q*_10_ along the steep slope

In contrast to soil respiration, the *Q*_10_ values were 13.2% greater at the upper slope position than at the lower slope position (Table 2), and thus provided the evidence to support our second hypothesis. The mechanism of the slope position effect on *Q*_10_ of soil respiration was complicated partly because the various respiration components (i.e., root respiration and microbial respiration) have different patterns of temperature responses [70–72]. Studies reported that no systematic differences were found in *Q*_10_ among roots differing in root biomass or root N concentration [73], and the microbial respiration *Q*_10_ was thus deduced to be the major driver of responses as far as this study is concerned. Therefore, the decreased *Q*_10_ downward on the slope may be largely attributable to the increased labile organic matter at the lower slope position (Table 3) according to the enzyme-activation theory that predicted that the substrate of the recalcitrant molecular structure was degraded with a higher *Q*_10_ [39].

### Potential role of microbial community in soil respiration and *Q*_10_

A notable finding of this study was the dramatic effect of slope positions on the soil microbial community (Table 4; Fig 3, S3 Text), which may potentially exert a large influence on the spatial variations of soil respiration and *Q*_10_ [26]. More specifically, the bacterial diversity was significantly greater at the lower slope position than at the upper slope position (Table 4). As increased bacterial diversity is always positively related to increased substrate C availability [74, 75], the fact that the alpha-diversity was higher at the lower slope position but lower at the upper slope position (Table 4) supports our expectation that the lower slope position had greater substrate C availability and thus higher soil microbial respiration. However, the fungal community in the surface soil changed from being Basidiomycota-dominant at the upper slope position to being Zygomycota-dominant at the lower slope position (Fig 3B, S3 Text). Many soil Zygomycota are copiotrophic and become abundant when labile substrate is available [76, 77], while Basidiomycota belong to oligotrophs and prefer nutrient-poor environments [78], leading to their contrasting distribution patterns at the upper and lower slope positions. Previous studies [79, 80] reported that the Zygomycota are specialized in the utilization of easily available C resources [81], characterized by higher maximal specific growth rates, higher metabolic quotients and higher maximum rates of soil respiration [68]. In contrast, most of the Basidiomycota are capable of decomposing more recalcitrant C [82], which inherently has lower decomposition rates [81]. Soil microbial communities shifted from oligotrophs to copiotrophs in the lower slope soil, reflecting greater microbial respiration rates. Therefore, a substantially greater soil respiration observed at the lower slope position was a product partly of copiotrophs replacing oligotrophs.

Apart from substrate quality, the variation in microbial respiration *Q*_10_ along the slope could also be attributed to shifts in microbial trophic strategy [75]. Changes in soil fungal community composition were closely linked to changes in soil organic carbon forms and microbial substrate utilization along the slope [83]. Specifically, in the lower slope position, shifts in fungal community composition (Zygomycota in place of Basidiomycota) suggest changes in microbial utilization to simple soluble C [84], possibly due to the large number of new and fresh C inputs [85, 86]. This pattern is in agreement with Graaff et al. [85], which reported that the large amounts of labile C addition changed the microbial community composition. Therefore, the explanation for the decreasing *Q*_10_ values down the slope could be the switch of Basidiomycota to Zygomycota and their utilization of the C source. This deduction was confirmed by the variations in enzyme activities (Table 3). The β-D-glucosidase activities significantly decreased at the lower slope position, which represents enzymes associated with complex organic matter (cellulose and lignin) breakdown [56]. This corresponds with the variation of Basidiomycota (Fig 3, S3 Text), which is responsible for the production of extracellular enzymes for the decomposition of lignin and celluloses [87, 88]. However, the increased activities of β-D-xylosidase and cellobiohydrolase suggest a shift of substrate utilization towards more labile [55] at the lower slope position. Furthermore, the fast-growing saprobic fungi Zygomycota that dominated the lower slope position (Table 4, Fig 3, S3 Text) mainly utilize simple soluble substrates [84] and are involved in ecological function through their metabolism without activation of enzyme activities. Therefore, it was speculated that the microbial respiration *Q*_10_ down the slope decreased due to the preferential use of nutrient-rich SOC of microbes when soil is rich in decomposable SOC [89]. Microbial substrate utilization downslope likely changed from relative more recalcitrant C, such as lignin and cellulose (upper slope position), to simple soluble C (lower slope position), which requires a lower activation energy for chemical and microbial decomposition [39, 90]. These are the evidence that erosional distribution of water and substrate leads to differential microbial utilization of SOC at different slope positions. This study also revealed that oligotrophic fungi responded positively with high *Q*_10_ values, while the copiotrophs showed a negative response. These results highlight the importance of fungal trophic strategy in the decomposition of SOC, and consequently the non-negligible relevance of fungal communities’ distribution in regulating soil respiration on sloping lands.

## Conclusions

In the hillslope grassland ecosystem of the Loess Plateau, contrasting responses of soil respiration (greater) and *Q*_10_ (lower) were observed at the lower slope position, which were attributed to the joint effects of more favorable soil moisture, greater root biomass, more organic substrate, predominance of labile C favoring Zygomycota and depressed enzyme activities. Apart from the less favorable soil moisture content and less labile substrate, the derived predominance of Basidiomycota and higher activities of enzymes associated with complex organic matter (cellulose and lignin) breakdown were also potential contributors to the lower soil respiration rates and greater *Q*_10_ at the upper slope position. Our findings suggest that the spatial variations of soil respiration and *Q*_10_ at different slope positions were not only affected by the potential effects of erosion-induced redistribution of water and nutrients along the slopes but also by the derived shifts in the microbial trophic strategy and substrate utilization. This casts a new light on our current understanding of the driving factors for the spatial variability of soil respiration and *Q*_10_ on steep slopes. Our observations also call for further investigations to identify the spatial distribution of specific microbial communities involved in soil C cycling along hillslopes and their relationships with soil water and nutrients, so as to help understand the responses of soil respiration on sloping land under future climate change.

## Supporting information

S1 Text Variations of air temperature, precipitation at the experimental site from 2014 to 2016. Data for Fig 1.
(PDF)

S2 Text Soil temperature, soil moisture and soil respiration rate at upper and lower slope positions over the study years. Data for Fig 2.
(PDF)

S3 Text Relative abundances of the dominant bacterial and fungal phyla at upper and lower slope positions. Data for Fig 3.
(PDF)
