# Supplementary material for: Spatial variations of soil respiration and temperature sensitivity along a steep slope of the semiarid Loess Plateau
Source: PLoS One. 2018 Apr 6;13(4):e0195400. doi: 10.1371/journal.pone.0195400 (PMC5889173; doi:10.1371/journal.pone.0195400)
Supplement: S1 Text — Data for Fig 1. (PDF) [file pone.0195400.s001.pdf]

Date	Air temperature/ ℃	Precipitation/mm
1/1/2014	-2.9625	0
2/1/2014	-0.9458	0
3/1/2014	-1.1208	0
4/1/2014	-0.8913	0
5/1/2014	-2.25	0
6/1/2014	-0.3042	0
7/1/2014	-0.8208	0
8/1/2014	-3.7375	0
9/1/2014	-6.7958	0
10/1/2014	-3.1292	0
11/1/2014	-2.4375	0
12/1/2014	-4.0542	0
13/1/2014	-6.0833	0
14/1/2014	-4.7333	0
15/1/2014	-5.5792	0
16/1/2014	-4.5917	0
17/1/2014	-2.9625	0
18/1/2014	-3.7333	0
19/1/2014	0.2417	0
20/1/2014	-2.7	0
21/1/2014	-3.7625	0
22/1/2014	-2.1167	0
23/1/2014	-0.3708	0
24/1/2014	3.7792	0
25/1/2014	0.8958	0
26/1/2014	-0.0375	0
27/1/2014	1.1125	0
28/1/2014	2.8875	0
29/1/2014	2.7875	0
30/1/2014	5.9917	0
31/1/2014	7.5174	0
1/2/2014	5.0917	0
2/2/2014	4.8875	0
3/2/2014	3.0917	0
4/2/2014	-2.925	0
5/2/2014	-6.3083	0
6/2/2014	-7.0333	0.8
7/2/2014	-6.3708	1.8
8/2/2014	-5.925	0.4
9/2/2014	-7.2458	1
10/2/2014	-9.0167	0
11/2/2014	-6.1708	0
12/2/2014	-5.4435	1
13/2/2014	-4.7917	0
14/2/2014	-4.4167	0
15/2/2014	-3.0208	0
16/2/2014	-1.9917	1.6

17/2/2014	-2.7833	1.8
18/2/2014	-1.2792	0
19/2/2014	-1.4958	0
20/2/2014	-0.175	0
21/2/2014	0.2375	0
22/2/2014	0.8292	0
23/2/2014	2.4625	0
24/2/2014	3.1	0.2
25/2/2014	2.7167	0.4
26/2/2014	3.7333	0
27/2/2014	2.1458	0
28/2/2014	0.7783	2.8
1/3/2014	0.9417	0
2/3/2014	1.3458	0
3/3/2014	0.7708	0
4/3/2014	2.6417	0
5/3/2014	1.2083	0.6
6/3/2014	1.1667	0
7/3/2014	0.4167	3.2
8/3/2014	1.6292	0
9/3/2014	4.0417	0
10/3/2014	7.475	0
11/3/2014	7.1875	0
12/3/2014	5.2417	0
13/3/2014	3.9292	0
14/3/2014	6.125	0
15/3/2014	9.675	0
16/3/2014	13.2292	0
17/3/2014	12.1958	0
18/3/2014	10.6875	0
19/3/2014	8.875	0
20/3/2014	6.2542	0
21/3/2014	4.8625	0
22/3/2014	8.7833	0
23/3/2014	10.1125	0
24/3/2014	11.5292	0
25/3/2014	13.3417	0
26/3/2014	15.2417	0
27/3/2014	12.7042	6
28/3/2014	9.8	0
29/3/2014	11.4833	0
30/3/2014	9.6174	13
31/3/2014	9.5	0
1/4/2014	9.5	0
2/4/2014	9.6208	0
3/4/2014	10.3	0
4/4/2014	9.2083	0
5/4/2014	10.9208	0

6/4/2014	10.8125	0
7/4/2014	11.8042	0
8/4/2014	14.1042	0
9/4/2014	14.4	0.2
10/4/2014	12.9	4.4
11/4/2014	10.925	14.6
12/4/2014	11.3083	0.8
13/4/2014	11.4583	0.2
14/4/2014	12.8083	0
15/4/2014	12.1875	0
16/4/2014	11.3542	3
17/4/2014	10.7917	0
18/4/2014	10.875	28
19/4/2014	10.1958	4
20/4/2014	10.3208	1.2
21/4/2014	10.3	14
22/4/2014	10.5125	0
23/4/2014	13.475	0
24/4/2014	14.7375	1.4
25/4/2014	5.6625	11.6
26/4/2014	7.5792	0
27/4/2014	9.9	0
28/4/2014	12.3875	0
29/4/2014	14.2542	0
30/4/2014	15.5542	0
1/5/2014	13.9042	0
2/5/2014	12.4417	0
3/5/2014	12.1042	0
4/5/2014	13.15	0
5/5/2014	10.7792	0
6/5/2014	13.5917	0.2
7/5/2014	18.3542	0
8/5/2014	20.125	0
9/5/2014	12.15	8
10/5/2014	8.4958	9.8
11/5/2014	11.5667	0
12/5/2014	11.5708	0
13/5/2014	15.1042	0
14/5/2014	13.3125	0
15/5/2014	13.5083	0
16/5/2014	14.0167	0
17/5/2014	14.175	0
18/5/2014	15.9875	0
19/5/2014	17.225	2.6
20/5/2014	17.6125	0
21/5/2014	19.45	0
22/5/2014	19.4542	0
23/5/2014	17.2875	8.4

24/5/2014	17.95	0.2
25/5/2014	16.6292	0
26/5/2014	17.1792	0
27/5/2014	19.6333	0
28/5/2014	0	0
29/5/2014	19.725	0
30/5/2014	19.8417	0
31/5/2014	18.9542	0
1/6/2014	18.9375	0
2/6/2014	20.0708	0
3/6/2014	20.0958	16.6
4/6/2014	18.9083	3.6
5/6/2014	19.6042	0
6/6/2014	18.8375	0
7/6/2014	16.7042	0
8/6/2014	19.2542	0
9/6/2014	20.7917	0
10/6/2014	20.9083	0
11/6/2014	21.9958	0
12/6/2014	18.3333	0.6
13/6/2014	16.6292	2.6
14/6/2014	17.2958	0.8
15/6/2014	19.8125	0
16/6/2014	22.7083	0
17/6/2014	21.8083	0
18/6/2014	22.4292	0
19/6/2014	19.7125	20
20/6/2014	18.6125	0
21/6/2014	20.3417	0
22/6/2014	21.2833	0
23/6/2014	21.6917	0
24/6/2014	20.0083	2
25/6/2014	17.8042	0.4
26/6/2014	19.4958	0.2
27/6/2014	22.7583	0
28/6/2014	19.0875	9
29/6/2014	20.4833	0.2
30/6/2014	21.9333	0
1/7/2014	21.8958	0
2/7/2014	21.9958	0
3/7/2014	21.3042	2.4
4/7/2014	21.5792	0
5/7/2014	22.6042	0
6/7/2014	24.2375	0
7/7/2014	25.5391	0
8/7/2014	22.75	1.4
9/7/2014	17.2583	13
10/7/2014	18.0833	1

11/7/2014	18.5	0
12/7/2014	20.5	0
13/7/2014	21.8083	0
14/7/2014	22.8	0
15/7/2014	23.3522	0
16/7/2014	24.7833	0
17/7/2014	24.9667	0
18/7/2014	25.3	0
19/7/2014	25.2958	1.6
20/7/2014	24.2042	2.4
21/7/2014	25.8	0
22/7/2014	24.8792	0
23/7/2014	19.3083	0
24/7/2014	20.7	0
25/7/2014	21.6792	0
26/7/2014	23.3	0
27/7/2014	24.8083	0
28/7/2014	26.1391	0
29/7/2014	26.6583	0
30/7/2014	27.6875	0
31/7/2014	27.2125	0
1/8/2014	25.725	0
2/8/2014	24.7667	0
3/8/2014	25.3	0
4/8/2014	23.9125	0
5/8/2014	23.6125	25.4
6/8/2014	17.8083	43.6
7/8/2014	16.7708	0
8/8/2014	15.7167	22.2
9/8/2014	17.4375	1.6
10/8/2014	18.9042	0.2
11/8/2014	21.3083	4.8
12/8/2014	18.0583	7.6
13/8/2014	19.1458	0.2
14/8/2014	18.5833	0
15/8/2014	19.0708	0
16/8/2014	19.6208	1.4
17/8/2014	19.1042	4.2
18/8/2014	20.6875	0
19/8/2014	19.4958	0
20/8/2014	18.8958	0
21/8/2014	19.3167	0
22/8/2014	19.3458	0
23/8/2014	20.3	3.4
24/8/2014	19.275	0.2
25/8/2014	18.7542	0
26/8/2014	19.4542	0
27/8/2014	19.1458	0

28/8/2014	20.1667	0
29/8/2014	20.2792	0
30/8/2014	18.9083	20.8
31/8/2014	18.45	0
1/9/2014	17.17	2.4
2/9/2014	17.3708	0.2
3/9/2014	16.1875	0
4/9/2014	17.075	0
5/9/2014	18.9125	0
6/9/2014	19.2	0
7/9/2014	19.075	0
8/9/2014	17.7542	10.2
9/9/2014	17.9167	6.6
10/9/2014	17.2292	15.6
11/9/2014	16.3	28.1
12/9/2014	18	0
13/9/2014	14.5	4.6
14/9/2014	13.1	20.9
15/9/2014	13.1	11.5
16/9/2014	12.25	15.1
17/9/2014	13.85	17.5
18/9/2014	15.8	0
19/9/2014	13.3	0
20/9/2014	14.35	0.3
21/9/2014	15.15	0
22/9/2014	14.9	7.6
23/9/2014	12.6	15.7
24/9/2014	13.3	0
25/9/2014	12.7	0
26/9/2014	14.2	0
27/9/2014	14.95	12
28/9/2014	14.9	19.4
29/9/2014	13	0
30/9/2014	16.35	0
1/10/2014	15.15	0
2/10/2014	14.5125	0
3/10/2014	14.125	0
4/10/2014	14.7333	0
5/10/2014	15.1125	0
6/10/2014	16.4542	0
7/10/2014	14.8417	0
8/10/2014	14.525	0
9/10/2014	14.9417	0
10/10/2014	15.6957	0
11/10/2014	14.3875	0
12/10/2014	16.0375	0
13/10/2014	15.1208	0
14/10/2014	11.8375	14.2

15/10/2014	8.4958	0
16/10/2014	9.0792	0
17/10/2014	8.0583	0
18/10/2014	8.0458	0
19/10/2014	8.725	0
20/10/2014	7.1125	0.4
21/10/2014	6.1875	0
22/10/2014	8.2167	0
23/10/2014	9.1	0
24/10/2014	9.0542	0
25/10/2014	6.25	0
26/10/2014	7.8333	0
27/10/2014	8.9083	0
28/10/2014	7.5167	0
29/10/2014	9.4125	0.8
30/10/2014	6.6208	13.2
31/10/2014	7.1	2.8
1/11/2014	7.95	1.2
2/11/2014	6.6083	0
3/11/2014	3.9708	0
4/11/2014	6.6958	2
5/11/2014	7.0792	0.2
6/11/2014	4.49	0
7/11/2014	4.6	0
8/11/2014	8.2783	0
9/11/2014	6.9292	3.6
10/11/2014	3.9958	0
11/11/2014	4.0833	0
12/11/2014	4.7708	0
13/11/2014	5.7667	0
14/11/2014	3.2125	0
15/11/2014	2.2708	0
16/11/2014	3.3625	0
17/11/2014	1.025	0
18/11/2014	0.7042	0
19/11/2014	1.0042	0
20/11/2014	2.6167	0
21/11/2014	1.7333	0
22/11/2014	1.6625	5.6
23/11/2014	0.4167	4
24/11/2014	0.7917	0.8
25/11/2014	0.56	0
26/11/2014	0.225	0
27/11/2014	-0.9583	0
28/11/2014	-2.3417	0
29/11/2014	-2.45	0
30/11/2014	1.5	0
1/12/2014	0.5375	0

2/12/2014	1.9667	0
3/12/2014	0.68	0
4/12/2014	0.15	0
5/12/2014	0.34	0
6/12/2014	0.12	0
7/12/2014	0.6458	0
8/12/2014	2.5083	0
9/12/2014	-2.2375	0
10/12/2014	-2.6792	0
11/12/2014	-3.9958	0
12/12/2014	-2.8333	0
13/12/2014	-2.9167	0
14/12/2014	-0.7333	0
15/12/2014	-0.2	0
16/12/2014	-1.9292	0
17/12/2014	-2.3917	0
18/12/2014	-3.6667	0
19/12/2014	-5.4348	0
20/12/2014	-2.6292	0
21/12/2014	-3.4208	0
22/12/2014	-4.7583	0
23/12/2014	-4.5792	0
24/12/2014	-2.4792	0
25/12/2014	-4	0
26/12/2014	-5.9208	0
27/12/2014	-8.7833	0
28/12/2014	-5.6458	0
29/12/2014	-6.5375	0
30/12/2014	-2.5958	0
31/12/2014	-1.3875	0
1/1/2015	-6.05	0
2/1/2015	0.26	0
3/1/2015	-2.14	0
4/1/2015	0.45	0
5/1/2015		
6/1/2015	-1.45	0
7/1/2015	-3.85	0
8/1/2015	-0.36	0
9/1/2015	-0.85	0
10/1/2015	-0.7	0
11/1/2015	-0.85	0
12/1/2015	-0.59	0
13/1/2015	-0.7	0
14/1/2015	-1.33	0
15/1/2015	-1.1	0
16/1/2015	-1.98	0
17/1/2015	-2.02	0
18/1/2015	-0.63	0

19/1/2015	-0.47	0
20/1/2015	2.37	0
21/1/2015	-0.91	0
22/1/2015	-1.72	0
23/1/2015	-0.77	0
24/1/2015	2.99	0
25/1/2015	-0.73	0
26/1/2015	0.72	0
27/1/2015	-1.52	1.2
28/1/2015	-5.75	0
29/1/2015	-5.63	2.6
30/1/2015	-7.36	0.4
31/1/2015	-6.13	1.2
1/2/2015	-5.31	0
2/2/2015	-3.73	0
3/2/2015	-2.65	0
4/2/2015	-1.75	0
5/2/2015	-3.71	0
6/2/2015	-3.05	0
7/2/2015	-0.66	0
8/2/2015	-2.49	0
9/2/2015	-1.5	0
10/2/2015	0	0
11/2/2015	4.07	0
12/2/2015	3.42	0
13/2/2015	3.39	0
14/2/2015	5.14	0
15/2/2015	6.17	0
16/2/2015	2.88	0
17/2/2015	4.14	0
18/2/2015	2.43	0
19/2/2015	1.03	1.2
20/2/2015	2.66	0
21/2/2015	3.38	0
22/2/2015	0.89	0
23/2/2015	2.58	0
24/2/2015	2.84	0
25/2/2015	4.37	0
26/2/2015	1.85	0
27/2/2015	0.63	4
28/2/2015	0.05	0
1/3/2015	-0.42	0
2/3/2015	2.13	0
3/3/2015	0.28	0
4/3/2015	-1.44	3.4
5/3/2015	-2.26	2.6
6/3/2015	-1.79	0
7/3/2015	2.59	0

8/3/2015	7.68	0
9/3/2015	3.61	0
10/3/2015		
11/3/2015	2.98	0
12/3/2015	6.74	0
13/3/2015	10.31	0
14/3/2015	9.81	0
15/3/2015	9.28	0
16/3/2015	10.32	0
17/3/2015	12.63	2
18/3/2015	7.01	5
19/3/2015	6.85	3.6
20/3/2015	7.15	0
21/3/2015	9.35	0
22/3/2015	7.82	0.2
23/3/2015	8.85	1.4
24/3/2015	6.13	7.2
25/3/2015	4.53	1.8
26/3/2015	4.55	0
27/3/2015	5.62	0
28/3/2015	8.95	0
29/3/2015	13.38	0
30/3/2015	14.94	0
31/3/2015	13.93	0
1/4/2015	11.46	15
2/4/2015	11.09	0.2
3/4/2015	8.8	1
4/4/2015	6.68	3.8
5/4/2015	7.83	3.2
6/4/2015	5.54	2.6
7/4/2015	5.52	0
8/4/2015	6.65	0
9/4/2015	8.82	0
10/4/2015	10.25	0
11/4/2015	12.88	0.4
12/4/2015		0
13/4/2015	7.44	0
14/4/2015	9.66	0
15/4/2015	15.83	0
16/4/2015	15.8	0
17/4/2015	13.07	0
18/4/2015	10.81	11.6
19/4/2015	11.72	6.2
20/4/2015	10.38	4.2
21/4/2015	11.86	8.4
22/4/2015	12.78	0
23/4/2015	13.3	0
24/4/2015	14	0.2

25/4/2015	13.82	0
26/4/2015	14.73	0
27/4/2015	16.39	0
28/4/2015	19.11	0
29/4/2015	17.57	0
30/4/2015	17.4	0.6
1/5/2015	12.44	10.4
2/5/2015	14.05	0
3/5/2015	14.75	0.2
4/5/2015	14.24	0
5/5/2015	15.18	0
6/5/2015	14.82	0
7/5/2015	13.58	0
8/5/2015	13.83	0
9/5/2015	17.48	0.4
10/5/2015	12.13	0.6
11/5/2015	10.56	0
12/5/2015	12.03	0
13/5/2015	17.89	0
14/5/2015	17.15	0
15/5/2015	17.3	0
16/5/2015		
17/5/2015	18.67	0
18/5/2015	21.32	0
19/5/2015	20.69	0
20/5/2015	18.38	2.2
21/5/2015	11.66	10.6
22/5/2015	14.67	0
23/5/2015	16.78	0
24/5/2015	17.16	0
25/5/2015	17.36	0
26/5/2015	18.4	0
27/5/2015	18.93	0
28/5/2015	16.36	6
29/5/2015	17.7	2
30/5/2015	17.08	20.4
31/5/2015	13.79	2.6
1/6/2015	16.8	5.4
2/6/2015	17.7	0
3/6/2015	15.49	14
4/6/2015	16.95	0.6
5/6/2015	19.4	0
6/6/2015	21.53	0
7/6/2015	18.62	0
8/6/2015	18.25	0
9/6/2015	20.16	0
10/6/2015	18.81	0.6
11/6/2015	19.44	0

12/6/2015	17.65	0
13/6/2015	19.62	0
14/6/2015	20.07	0
15/6/2015	16.37	2.8
16/6/2015	16.9	0
17/6/2015	18.59	0
18/6/2015	22.59	0
19/6/2015	21.14	1
20/6/2015	18.25	0.2
21/6/2015	18.1	0.2
22/6/2015	19.05	0
23/6/2015	16.98	34.4
24/6/2015	17.63	3.4
25/6/2015	19.34	0.8
26/6/2015	18.42	3.2
27/6/2015	17.63	5.4
28/6/2015	17.72	9.6
29/6/2015	20.37	12
30/6/2015	23.32	0
1/7/2015	23.15	0
2/7/2015	22.31	0
3/7/2015	21.18	0
4/7/2015	19.3	0
5/7/2015	20.11	0
6/7/2015	20.1	0
7/7/2015	21.32	0
8/7/2015		
9/7/2015	20.61	0
10/7/2015	19.85	0
11/7/2015		
12/7/2015	23.27	0
13/7/2015	25.23	0
14/7/2015	22.12	0
15/7/2015	21.78	0
16/7/2015	19.71	4.4
17/7/2015	19.86	1.6
18/7/2015	20.75	0.2
19/7/2015		
20/7/2015	20.07	0
21/7/2015	21.65	0
22/7/2015	22.19	0
23/7/2015		
24/7/2015		
25/7/2015	22.67	0
26/7/2015	23.44	0
27/7/2015	23.7	0
28/7/2015	25.74	0
29/7/2015	25.41	0

30/7/2015	25.45	0
31/7/2015	25.96	0
1/8/2015	26.74	0
2/8/2015	25.13	2.4
3/8/2015	18.95	7.6
4/8/2015	17.03	4.6
5/8/2015	17.8	0
6/8/2015	21.86	0
7/8/2015	21.48	0
8/8/2015	21.45	0.6
9/8/2015	19.88	0.8
10/8/2015	20.38	1.6
11/8/2015	21.17	12.2
12/8/2015	19.22	86.6
13/8/2015	20.57	0
14/8/2015	19.38	1.2
15/8/2015	18.76	0
16/8/2015	19.73	0.4
17/8/2015	18.33	0.8
18/8/2015	17.48	0
19/8/2015	19.2	0
20/8/2015	20.36	0
21/8/2015	19.56	0
22/8/2015	19.85	0
23/8/2015	19.23	0
24/8/2015	17.58	0
25/8/2015	18.22	0
26/8/2015	19.29	10.4
27/8/2015	18.82	0.2
28/8/2015	19.46	0
29/8/2015	19.39	0
30/8/2015	20.27	0
31/8/2015	19.5	0.2
1/9/2015	19.37	0
2/9/2015	18.51	0
3/9/2015	17.35	11.6
4/9/2015	19.56	5.6
5/9/2015	20.77	0
6/9/2015	19.41	0
7/9/2015	18.1	0.4
8/9/2015	16.93	6.8
9/9/2015	16.19	7
10/9/2015	15.68	8.2
11/9/2015	15.21	0
12/9/2015	14.4	0
13/9/2015	15.33	0
14/9/2015	14.96	0
15/9/2015	16.13	0

16/9/2015	16.02	0
17/9/2015	15.2	2
18/9/2015	15.98	0
19/9/2015	15.26	0
20/9/2015	15.25	0
21/9/2015	16.2	0
22/9/2015	15.85	7.6
23/9/2015	12	0
24/9/2015	13.25	5
25/9/2015	14.42	0
26/9/2015	15.42	0
27/9/2015	15.56	0
28/9/2015	14.09	1.2
29/9/2015	13.68	1.8
30/9/2015	12.06	4.4
29/9/2015	8.93	0
30/9/2015	6.3	0
1/10/2015	8.18	0
2/10/2015	11.3	0
3/10/2015	11.16	0
4/10/2015	11.61	3
5/10/2015	13.6	0
6/10/2015	9.65	3.4
7/10/2015	8.14	0
8/10/2015	8.83	0
9/10/2015	7.37	0
10/10/2015	8.48	0
11/10/2015	8.71	1
12/10/2015	10.93	0
13/10/2015	11.48	0
14/10/2015	13.2	0
15/10/2015	15.22	0
16/10/2015	12.15	0
17/10/2015	10.55	0
18/10/2015	12.42	0
19/10/2015	12.82	0
20/10/2015	9.76	0.2
21/10/2015	10.6	0.2
22/10/2015	10.46	19
23/10/2015	9.58	6.6
24/10/2015	6.33	5.6
25/10/2015	3.84	0
26/10/2015	7.48	0
27/10/2015	6.97	0
28/10/2015	6.32	2.4
29/10/2015	2.98	2.2
30/10/2015	3.05	0
31/10/2015	4.26	0

1/11/2015	8.57	0
2/11/2015	9.42	0.4
3/11/2015	7.41	5.6
4/11/2015	6.64	8.6
5/11/2015	4.59	0.2
6/11/2015	2.44	0
7/11/2015	2.78	0
8/11/2015	6.6	0
9/11/2015	5.68	0
10/11/2015	4.4	1.8
11/11/2015	4.85	0
12/11/2015	4.96	0
13/11/2015	6.6	0
14/11/2015	4.29	0
15/11/2015	5.64	2.4
16/11/2015	5.18	0
17/11/2015	5.38	0
18/11/2015	3.33	0
19/11/2015	4.93	0.2
20/11/2015	5.8	0
21/11/2015	5.5	3.8
22/11/2015	2.66	4.4
23/11/2015	0.51	0
24/11/2015	-0.14	0
25/11/2015	1.3	0
26/11/2015	1.75	0
27/11/2015	2.75	0
28/11/2015	2.95	0
29/11/2015	3.96	0
30/11/2015	2.37	0
1/12/2015	-1.23	0
2/12/2015	-2.62	0
3/12/2015	-0.2	0
4/12/2015	-0.91	0
5/12/2015	0.89	0
6/12/2015	-1.1	0
7/12/2015	-0.02	0
8/12/2015	0.27	0
9/12/2015	-0.31	0.4
10/12/2015	-1.02	4.6
11/12/2015	-1.78	2.6
12/12/2015	-5.09	0
13/12/2015	-5.48	0
14/12/2015	-7.9	0
15/12/2015	-10.19	0
16/12/2015	-5.84	0
17/12/2015	-3.3	0
18/12/2015	-5.94	0

19/12/2015	-2.79	0
20/12/2015	-1.52	0
21/12/2015	-4.84	0
22/12/2015	-2.25	0
23/12/2015	-2.18	0
24/12/2015	-1.86	0
25/12/2015	-1.42	0
26/12/2015	-3.25	0
27/12/2015	-1.91	0
28/12/2015	-1.41	0
29/12/2015	-0.46	0
30/12/2015	3.43	0
31/12/2015	0.77	0
1/1/2016	3.43	0
2/1/2016	0.77	0
3/1/2016	2.75	0
4/1/2016	2.93	0
5/1/2016	0.58	0
6/1/2016	-2.72	0
7/1/2016	-0.94	0
8/1/2016	-4.07	0
9/1/2016	-2.53	0
10/1/2016	-2.17	0
11/1/2016	-2.79	0
12/1/2016	-4.35	1.6
13/1/2016	-8.02	0
14/1/2016	-4.95	0
15/1/2016	-4.95	0
16/1/2016	-0.64	0
17/1/2016	-1.32	0
18/1/2016	-3.68	0
19/1/2016	-5.49	0
20/1/2016	-4.64	1
21/1/2016	-4.25	0
22/1/2016	-7.9	0
23/1/2016	-11.24	0
24/1/2016	-14.03	0
25/1/2016	-12.13	0
26/1/2016	-8.51	0
27/1/2016	-7.3	0
28/1/2016	-4.03	0
29/1/2016	-4.55	0
30/1/2016	-3.23	0
31/1/2016	-5.6	0
1/2/2016	-7.38	0
2/2/2016	-6.42	0
3/2/2016	-3.84	0
4/2/2016	-0.32	0

5/2/2016	-2.5	0
6/2/2016	-3.78	0
7/2/2016	3.65	0
8/2/2016	2.39	0
9/2/2016	2.82	0
10/2/2016	3.68	0
11/2/2016	3.4	0
12/2/2016	-1.14	0.8
13/2/2016	-6.77	0
14/2/2016	-7.59	0.2
15/2/2016	-7.8	0
16/2/2016	-3.24	0
17/2/2016	-1.31	0
18/2/2016	1.03	0
19/2/2016	0.48	0
20/2/2016	-1.2	0
21/2/2016	-2.47	0
22/2/2016	-0.73	0
23/2/2016	1.32	0
24/2/2016	1.89	0
25/2/2016	3.58	0
26/2/2016	4.53	0
27/2/2016	7.8	0
28/2/2016	6.79	0
29/2/2016	3	0
1/3/2016	4.7	0
2/3/2016	7.99	0
3/3/2016	9.07	0
4/3/2016	7.01	0
5/3/2016	2.95	0
6/3/2016	7.29	0
7/3/2016	6.96	0.4
8/3/2016	3.58	0
9/3/2016	-0.28	5.4
10/3/2016	-4.2	0
11/3/2016	1.25	0
12/3/2016	4.13	0
13/3/2016	5.5	0
14/3/2016	5.31	0
15/3/2016	8.97	0
16/3/2016	7.95	0
17/3/2016	9.37	0
18/3/2016	9.87	0
19/3/2016	13.06	0
20/3/2016	11.84	0
21/3/2016	7.75	2.8
22/3/2016	8.03	1
23/3/2016	7.04	3.8

24/3/2016	6.08	2.8
25/3/2016	5.2	0.8
26/3/2016	4.95	0
27/3/2016	6.13	0
28/3/2016	10.7	0
29/3/2016	12.29	0
30/3/2016	12.07	0
31/3/2016	15.18	0
1/4/2016	16.36	0
2/4/2016	15.39	0
3/4/2016	9.17	0
4/4/2016	6.8	1.8
5/4/2016	7.75	0.4
6/4/2016	11.16	1.4
7/4/2016	13.33	0
8/4/2016	12.8	1.2
9/4/2016	11.02	0.4
10/4/2016	13.83	0
11/4/2016	15.59	0.2
12/4/2016	13.64	0
13/4/2016	11.25	0
14/4/2016	12.48	0
15/4/2016	11.13	19.2
16/4/2016	9.6	0
17/4/2016	10.25	0.2
18/4/2016	11.57	0
19/4/2016	13.53	0
20/4/2016	15.88	0
21/4/2016	14.75	0
22/4/2016	13.74	0
23/4/2016		
24/4/2016	14.06	0
25/4/2016	13.18	2.6
26/4/2016	14.9	0
27/4/2016	16.3	0
28/4/2016	15.58	0
29/4/2016	17.18	0
30/4/2016	19.06	0
1/5/2016	19.47	0
2/5/2016	12.51	8
3/5/2016	12.46	0
4/5/2016	17.73	0
5/5/2016	20.86	0
6/5/2016	14.96	0
7/5/2016	8.31	7.2
8/5/2016	8.85	0
9/5/2016	14.47	0
10/5/2016	16.95	0

11/5/2016	21.13	0
12/5/2016	14.45	0
13/5/2016	6.07	0.8
14/5/2016	9.14	11.4
15/5/2016	12.21	0
16/5/2016	12.56	0
17/5/2016	16.63	0
18/5/2016	18.28	0
19/5/2016	17.4	0
20/5/2016	16.58	0
21/5/2016	17.19	0
22/5/2016	16.19	0.8
23/5/2016	13.42	1.8
24/5/2016	15.01	0
25/5/2016	12.67	1.8
26/5/2016	11.34	0
27/5/2016	10.3	5.2
28/5/2016	13.57	0
29/5/2016	16.38	0
30/5/2016	18	0
31/5/2016	18.6	0.6
1/6/2016	15.3	4
2/6/2016	12.61	11.2
3/6/2016	16.59	0.8
4/6/2016	19.31	1
5/6/2016	21.09	0
6/6/2016	19.89	0
7/6/2016	20.43	1.8
8/6/2016	18.69	0
9/6/2016	17.62	6.2
10/6/2016	20.71	0
11/6/2016	21.17	0.4
12/6/2016	18.45	0
13/6/2016	20.88	0
14/6/2016	21.96	8.8
15/6/2016	19.47	0
16/6/2016	18.52	0
17/6/2016	22.74	0
18/6/2016	24.06	0
19/6/2016	23.64	5.2
20/6/2016	22.96	0
21/6/2016	22.86	0
22/6/2016	22.74	0
23/6/2016	19.67	11
24/6/2016	17.84	0
25/6/2016	20.63	0
26/6/2016	19.85	0.8
27/6/2016	19.09	0.2

28/6/2016	22.83	0
29/6/2016	24.51	0
30/6/2016	23.7	0.2
1/7/2016	21.88	0
2/7/2016	22.39	0
3/7/2016	24.96	0
4/7/2016	23.64	0
5/7/2016	23.75	0
6/7/2016	23.4	0
7/7/2016	22.65	0
8/7/2016	24.11	0
9/7/2016	25.35	0
10/7/2016	24.89	0
11/7/2016	23.46	30.6
12/7/2016	22.65	0.2
13/7/2016	21.53	1.2
14/7/2016	18.03	11
15/7/2016	19.33	2.6
16/7/2016	22.62	0.8
17/7/2016	21.83	0
18/7/2016	20.5	44.2
19/7/2016		
20/7/2016	22.3	0
21/7/2016	23.19	0
22/7/2016	20.7	6.2
23/7/2016	22.08	0
24/7/2016	23.51	28
25/7/2016	22.69	0.2
26/7/2016	20.71	0.6
27/7/2016	22.36	0.2
28/7/2016	24.8	0
29/7/2016	25.21	0
30/7/2016	24.59	0
31/7/2016	23.13	0
1/8/2016	23.18	2.4
2/8/2016	23.37	0
3/8/2016	24.12	0
4/8/2016	23.07	0
5/8/2016	22.65	0
6/8/2016	21.08	0
7/8/2016	22.18	0
8/8/2016	22.22	0
9/8/2016	22.64	0
10/8/2016	23.96	0
11/8/2016	24.75	0
12/8/2016	26.08	0
13/8/2016	26.18	0
14/8/2016	27.75	0

15/8/2016	27.56	0
16/8/2016	26.98	0
17/8/2016	26.61	0
18/8/2016	25.38	0
19/8/2016	26.13	0
20/8/2016	27.01	0
21/8/2016	26.01	0
22/8/2016	25.63	0
23/8/2016	24.06	0
24/8/2016	24.87	0
25/8/2016	18.89	144.6
26/8/2016	18.56	0
27/8/2016	18.2	0
28/8/2016	18.8	0
29/8/2016	16.72	0.4
30/8/2016	17.62	0.2
31/8/2016	19.07	0
1/9/2016	19.25	0
2/9/2016	18.96	0
3/9/2016	18.46	0.8
4/9/2016	17.98	0
5/9/2016	18.53	0
6/9/2016	16.85	2.8
7/9/2016	16.03	0.2
8/9/2016	17.49	0
9/9/2016	15.8	2
10/9/2016	15.15	0.6
11/9/2016	16.75	0
12/9/2016	15.21	2.6
13/9/2016	15.81	0
14/9/2016	16.15	0
15/9/2016	16.16	0
16/9/2016	16.35	0
17/9/2016	17.38	1
18/9/2016	16.42	14.6
19/9/2016	16.45	0.2
20/9/2016	13.45	0.2
21/9/2016	14.92	0
22/9/2016	16.36	0.2
23/9/2016	18.12	0
24/9/2016	16.84	2.6
25/9/2016	15.04	0
26/9/2016	15.53	0
27/9/2016	15.28	0
28/9/2016	13.61	0
29/9/2016	12.93	0
30/9/2016	15.21	0
1/10/2016	17.1	0

2/10/2016		
3/10/2016	19.1	0
4/10/2016	15	0.4
5/10/2016	7.4	1.2
6/10/2016	9.7	8
7/10/2016	12.7	7.8
8/10/2016	11.7	0
9/10/2016	9.8	3.8
10/10/2016	8.9	0
11/10/2016	10.3	0
12/10/2016	10.8	0.2
13/10/2016	9.9	0
14/10/2016	10.8	0.4
15/10/2016	10.2	3
16/10/2016	11.7	0
17/10/2016	10.7	0
18/10/2016	11.4	0
19/10/2016	11.7	0
20/10/2016	16.6	0
21/10/2016	12.3	2.8
22/10/2016	9.4	0.8
23/10/2016	8.3	2.8
24/10/2016	8.9	9
25/10/2016	6.6	0.2
26/10/2016	8.8	4.2
27/10/2016	6.6	6.4
28/10/2016	3.2	0
29/10/2016	4.3	0
30/10/2016	4.8	0
31/10/2016	5.9	0
1/11/2016	3.05	0
2/11/2016	4.26	0
3/11/2016	8.57	0
4/11/2016	9.42	0.4
5/11/2016	7.41	5.6
6/11/2016	6.64	8.6
7/11/2016	4.59	0.2
8/11/2016	2.44	0
9/11/2016	2.78	0
10/11/2016	6.6	0
11/11/2016	5.68	0
12/11/2016	4.4	1.8
13/11/2016	4.85	0
14/11/2016	4.96	0
15/11/2016	6.6	0
16/11/2016	4.29	0
17/11/2016	5.64	2.4
18/11/2016	5.18	0

19/11/2016	5.38	0
20/11/2016	3.33	0
21/11/2016	4.93	0.2
22/11/2016	5.8	0
23/11/2016	5.5	3.8
24/11/2016	2.66	4.4
25/11/2016	0.51	0
26/11/2016	-0.14	0
27/11/2016	1.3	0
28/11/2016	1.75	0
29/11/2016	2.75	0
30/11/2016	2.95	0
1/12/2016	3.96	0
2/12/2016	2.37	0
3/12/2016	-1.23	0
4/12/2016	-2.62	0
5/12/2016	-0.2	0
6/12/2016	-0.91	0
7/12/2016	0.89	0
8/12/2016	-1.1	0
9/12/2016	-0.02	0
10/12/2016	0.27	0
11/12/2016	-0.31	0.4
12/12/2016	-1.02	4.6
13/12/2016	-1.78	2.6
14/12/2016	-5.09	0
15/12/2016	-5.48	0
16/12/2016	-7.9	0
17/12/2016	-10.19	0
18/12/2016	-5.84	0
19/12/2016	-3.3	0
20/12/2016	-5.94	0
21/12/2016	-2.79	0
22/12/2016	-1.52	0
23/12/2016	-4.84	0
24/12/2016	-2.25	0
25/12/2016	-2.18	0
26/12/2016	-1.86	0
27/12/2016	-1.42	0
28/12/2016	-3.25	0
29/12/2016	-1.91	0
30/12/2016	-1.41	0
31/12/2016	-0.46	0
1/1/2017		
2/1/2017		
3/1/2017		
4/1/2017		
5/1/2017		

6/1/2017		
7/1/2017	-0.02	1.8
8/1/2017	-0.05	0
9/1/2017	-0.13	0.4
10/1/2017	-0.03	0
11/1/2017	-0.08	0
12/1/2017	-0.49	0
13/1/2017	-1.15	0
14/1/2017	-1.29	0
15/1/2017	-0.73	0
16/1/2017	-1.69	0
17/1/2017	-1.98	0
18/1/2017	-0.98	0
19/1/2017	-0.98	0
20/1/2017	-1.94	0
21/1/2017	-2.25	0
22/1/2017	-1.64	0
23/1/2017	-1.53	0
24/1/2017	-0.71	0
25/1/2017	-0.7	0
26/1/2017	-0.2	0
27/1/2017	-0.36	0
28/1/2017	-0.12	0
29/1/2017	-0.07	0
30/1/2017	-0.2	0
31/1/2017	-0.65	0
1/2/2017	-1.45	0
2/2/2017	-0.25	0
3/2/2017	-0.22	0
4/2/2017	-0.15	0
5/2/2017	-0.03	0
6/2/2017	0	2.2
7/2/2017	0	2.4
8/2/2017	0.05	0.4
9/2/2017	-0.04	0
10/2/2017	-0.22	0
11/2/2017	-0.29	0
12/2/2017	-0.03	0
13/2/2017	0.09	0
14/2/2017	1.81	0
15/2/2017	2.59	0
16/2/2017	2.82	0
17/2/2017	3.89	0
18/2/2017	3.21	0
19/2/2017	3.94	0
20/2/2017	3.65	0
21/2/2017	1.17	8.4
22/2/2017	0.63	0

23/2/2017	1.16	0
24/2/2017	1.76	0
25/2/2017	2.6	0
26/2/2017	3.04	0
27/2/2017	3.34	0
28/2/2017	3.89	0
1/3/2017	4.07	0
2/3/2017	3.16	0
3/3/2017	4.25	0
4/3/2017	4.12	0
5/3/2017	3.98	0
6/3/2017	3.44	0
7/3/2017	3.41	0
8/3/2017	3.74	0
9/3/2017	3.64	0
10/3/2017	4.49	0
11/3/2017	4.37	3.2
12/3/2017	3.32	20.6
13/3/2017	1.85	12.6
14/3/2017	1.55	0
15/3/2017	1.4	1.8
16/3/2017	1.33	0
17/3/2017	3.24	0
18/3/2017	4.3	0
19/3/2017	4.72	1.2
20/3/2017	5.45	0
21/3/2017	4.73	0
22/3/2017	5.97	1.6
23/3/2017	5.67	0.8
24/3/2017	4.29	0
25/3/2017	4.98	0
26/3/2017	5.87	0
27/3/2017	7.41	0
28/3/2017	8.52	0
29/3/2017	7.03	0.4
30/3/2017	8.76	5.2
31/3/2017	8.99	0
1/4/2017	8.83	0
2/4/2017	8.73	0
3/4/2017	9.19	2.4
4/4/2017	11.15	1.6
5/4/2017	12.1	0
6/4/2017	13.1	0
7/4/2017	13.23	0
8/4/2017	11.57	2
9/4/2017	10.69	8.2
10/4/2017	10.63	5.4
11/4/2017	12.05	0.2

12/4/2017	11.66	0
13/4/2017	12.7	3
14/4/2017	13.22	0
15/4/2017	12.11	0
16/4/2017	11.34	0
17/4/2017	11.76	0
18/4/2017	14.03	0
19/4/2017	12.35	2
20/4/2017	10.8	0
21/4/2017	10.95	0
22/4/2017	11.26	1
23/4/2017	12.32	0
24/4/2017	13.51	0
25/4/2017	14.73	0
26/4/2017	12.43	0
27/4/2017	12.4	0
28/4/2017	12.86	0
29/4/2017	13.46	0
30/4/2017	13.86	0
1/5/2017	15.1	0
2/5/2017	14.46	7.6
3/5/2017	13.27	15.8
4/5/2017	14.19	0
5/5/2017	13.72	0
6/5/2017	13.03	0
7/5/2017	12.06	0
8/5/2017	12.2	0
9/5/2017	14.08	0
10/5/2017	14.04	0
11/5/2017	14.63	0
12/5/2017	15	0
13/5/2017	14.87	0
14/5/2017	14.95	0.4
15/5/2017	13.57	0.8
16/5/2017	14.79	0
17/5/2017	15.65	0
18/5/2017	16.73	7.2
19/5/2017	17.15	2.2
20/5/2017	17.35	0
21/5/2017	18.12	0
22/5/2017	16.18	17.6
23/5/2017	14.45	0
24/5/2017	15.06	0
25/5/2017	15.03	0
26/5/2017	15.89	0
27/5/2017	16.84	0
28/5/2017	17.34	0
29/5/2017	16.35	0.8

30/5/2017	16.03	0.2
31/5/2017	17.2	0
1/6/2017	18.41	0
2/6/2017	18.14	0
3/6/2017	17.45	5
4/6/2017	15.99	43.4
5/6/2017	15.81	25.8
6/6/2017	16.99	0
7/6/2017	17.19	3.4
8/6/2017	17.54	1.6
9/6/2017	17.79	0.4
10/6/2017	17.12	0.2
11/6/2017	19.02	0
12/6/2017	20.58	0
13/6/2017	21.52	0
14/6/2017	21.79	0
15/6/2017	21.92	0
16/6/2017	23.12	0
17/6/2017	23.16	0
18/6/2017	23.35	0
19/6/2017	23.85	14.6
20/6/2017	22.51	0.2
21/6/2017	21.9	11
22/6/2017	22.53	1.4
23/6/2017	21.53	0.2
24/6/2017	20.83	0
25/6/2017	21.74	0
26/6/2017	22.54	0
27/6/2017	23.1	0
28/6/2017	23.29	0
29/6/2017	23	0
30/6/2017	22.66	0
1/7/2017	23.15	0
2/7/2017	23.85	0
3/7/2017	24.13	0
4/7/2017	23.5	0.2
5/7/2017	22.34	5.2
6/7/2017	22.88	1.6
7/7/2017	22.63	0
8/7/2017	22.27	0
9/7/2017	23.53	0
10/7/2017	23.73	0
11/7/2017	24.2	0
12/7/2017	25.07	0
13/7/2017	25.45	0
14/7/2017	25.8	0
15/7/2017	24.85	15.4
16/7/2017	24.78	0

17/7/2017	24.26	4.8
18/7/2017	25.07	0
19/7/2017	26.13	0
20/7/2017	26.87	0
21/7/2017	27.77	0
22/7/2017	28.2	0
23/7/2017	28.1	0
24/7/2017	28.15	0
25/7/2017	27.77	0
26/7/2017	27.63	0
27/7/2017	26.98	2.4
28/7/2017	23.13	11.4
29/7/2017	23.49	0
30/7/2017	24.94	0
31/7/2017	24.95	0
1/8/2017	25.14	0
2/8/2017	26.34	0
3/8/2017	26.63	0
4/8/2017	26.36	0
5/8/2017	26.2	0
6/8/2017	25.44	2
7/8/2017	22.14	10.8
8/8/2017	22.23	0.2
9/8/2017	24.01	0
10/8/2017	23.88	0
11/8/2017	24.74	0
12/8/2017	25.17	1.8
13/8/2017	23.86	0.2
14/8/2017	23.34	0
15/8/2017	24.26	0
16/8/2017	25.55	0
17/8/2017	26.05	0
18/8/2017	24.11	19.4
19/8/2017	22.72	2
20/8/2017	22.4	20.2
21/8/2017	20.99	9.8
22/8/2017	20.56	40.4
23/8/2017	20.15	0
24/8/2017	21.52	0
25/8/2017	20.66	0
26/8/2017	19.16	2.6
27/8/2017	18.78	2
28/8/2017	18.48	4.4
29/8/2017	17.48	16.6
30/8/2017	16.63	17
31/8/2017	17.73	0
1/9/2017	18.25	0
2/9/2017	18.48	0

3/9/2017	19.46	0
4/9/2017	18.92	5.8
5/9/2017	17.93	6.2
6/9/2017	17.65	0
7/9/2017		
8/9/2017	19.24	0
9/9/2017	18.33	1.4
10/9/2017	18.81	0.4
11/9/2017		
12/9/2017	17.77	0
13/9/2017	18.18	0
14/9/2017	18.41	0
15/9/2017		
16/9/2017	18.49	3.8
17/9/2017	18.47	0
18/9/2017	18.6	0.2
19/9/2017	18.77	0
20/9/2017	18.6	0
21/9/2017	17.76	0
22/9/2017	16.07	0.2
23/9/2017	15.63	0.2
24/9/2017	16.76	0
25/9/2017	17.95	3.6
26/9/2017	17.19	2.2
27/9/2017	17.12	1
28/9/2017	17.28	0.2
29/9/2017	18.67	0
30/9/2017	17.04	2.2
1/10/2017	17.25	0.4
2/10/2017	16.93	0.2
3/10/2017	13.8	31.4
4/10/2017	13.19	6.2
5/10/2017	13.23	0
6/10/2017	13.95	0
7/10/2017	14.55	1.2
8/10/2017	14.6	15.6
9/10/2017	12.12	10.4
10/10/2017	9.57	10
11/10/2017	8.68	1.6
12/10/2017	9.82	0
13/10/2017	11.01	0.6
14/10/2017	11.57	3.4
15/10/2017	11.53	0.6
16/10/2017	11.03	2.6
17/10/2017	10.54	0.8
18/10/2017	11.26	0
19/10/2017	11.8	0
20/10/2017	11.61	0

21/10/2017	11.1	0
22/10/2017	10.71	0
23/10/2017	12.2	0
24/10/2017	12.31	0
25/10/2017	11.86	4.2
26/10/2017	10.75	0
27/10/2017	10.2	0
28/10/2017	10.33	0
29/10/2017	9.52	0
30/10/2017	9.71	1.2
31/10/2017	10.27	2.2
1/11/2017	10.92	0.4
2/11/2017	10.05	0
3/11/2017	9.68	0
4/11/2017	8.79	0
5/11/2017	9.21	0
6/11/2017	8.75	0
7/11/2017	9.09	0
8/11/2017	8.11	0
9/11/2017	8.54	0
10/11/2017	7.67	0
11/11/2017	5.6	0
12/11/2017	5.78	0
13/11/2017	5.57	0
14/11/2017	5.58	0
15/11/2017	5.15	0
16/11/2017	4.99	0
17/11/2017	6.3	0
18/11/2017	5.14	0
19/11/2017	4.7	1
20/11/2017	3.13	0
21/11/2017	4.46	0
22/11/2017	3.15	0
23/11/2017	2.23	0
24/11/2017	1.53	0
25/11/2017	1.05	0
26/11/2017	0.77	0
27/11/2017	1.32	0
28/11/2017	1.58	0
29/11/2017	2.47	0
30/11/2017	1.58	0
