# Supplementary material for: Spatial variations of soil respiration and temperature sensitivity along a steep slope of the semiarid Loess Plateau
Source: PLoS One. 2018 Apr 6;13(4):e0195400. doi: 10.1371/journal.pone.0195400 (PMC5889173; doi:10.1371/journal.pone.0195400)
Supplement: S2 Text — Data for Fig 2. (PDF) [file pone.0195400.s002.pdf]

Date	Soil temperature_Upper/ ℃	SE1	Soil temperature_Lower/ ℃	SE2
8/5/2014	11.8667	0.1247	12.5833	0.2694
12/5/2014	14.1	0.9327	15	1.778
15/5/2014	16.3	1.6649	18.1667	1.4496
22/5/2014	20.7667	1.0405	20.5	0.8177
23/5/2014	16.9167	0.2181	17.6667	0.5458
25/5/2014	17.3	0.6779	16.7667	0.7355
15/6/2014	23.55	0.8499	23.3333	0.8585
20/6/2014	20.6667	0.5574	20.7167	0.6591
30/6/2014	23.6	0.5275	23.5167	0.9595
11/7/2014	24.4167	6.5276	26.15	0.3295
12/7/2014	24.7	0.97	25.9833	0.3355
13/7/2014	23.8167	1.9429	25.3167	0.6074
14/7/2014	21.4167	0.7489	23.1833	1.038
15/7/2014	25.9667	0.2329	26.7333	0.6402
16/7/2014	26.65	1.7836	26.85	0.6223
23/7/2014	27.05	0.9092	28.8667	0.4686
27/7/2014	28.8333	#DIV/0!	26.6	0.0408
10/8/2014	22.2	0.1075	22.1167	0.3477
14/8/2014	21.9667	0.7939	22.2167	0.6071
24/8/2014	21.5333	1.2246	22.6333	0.8708
20/9/2014	18.7833	0.3472	19.8	0.2959
26/9/2014	18.65	1.0626	18.6	0.8698
5/10/2014	17	0.8446	17.6333	0.6361
17/10/2014	13.5333	0.3107	14.6	0.6465
31/10/2014	9.1	0.2652	9.7167	0.7234
24/12/2014	2.4333	0.5582	2.2	1.1958
10/3/2015	8.8167	0.6904	7.6667	1.0922
28/3/2015	11.3167	0.928	11.2333	0.5457
10/4/2015	13.5333	0.5397	11.9833	0.8942
23/4/2015	15.2833	0.3736	14.7833	0.7321
8/5/2015	18.6667	1.1435	18.6333	0.3215
18/5/2015	23.1667	0.1225	22.7833	0.0408
6/6/2015	23.5833	0.148	25.0667	0.4057
10/6/2015	19.8333	0.9675	22.6667	0.7014
24/6/2015	21.6333	0.9025	22.0833	0.4631
27/6/2015	23.7833	0.4111	24.8167	0.3357
1/7/2015	25.7167	0.1856	26.2	0.1202
5/7/2015	30.4167	0.6969	30.45	0.3496
8/7/2015	25.3167	0.949	25.4833	1.1971
14/7/2015	25.5667	1.3775	27.2	0.8
26/7/2015	27.35	1.3676	30.05	1.3821
12/8/2015	22.4	0.1581	22.65	0.1011
22/8/2015	23.7833	0.0675	24.65	0.2852
26/8/2015	23.8167	1.075	26.5	1.1211
6/9/2015	22.0667	1.5482	24.2667	0.5645
13/9/2015	18.6667	0.6491	20.1833	0.3961

16/9/2015	20.35	0.557	22.3667	0.4512
17/9/2015	18.5667	0.3266	19.5667	0.5029
19/9/2015	19.4	0.6812	20.45	0.6925
26/9/2015	18.88	0.4079	19.775	1.4177
14/10/2015	13.9333	2.3032	13.8667	1.6992
7/12/2015	2.4333	0.2495	2.2	0.0789
13/3/2016	4.7833	0.9372	3.7167	0.9113
14/4/2016	16.2167	0.6467	14.1667	0.3559
17/4/2016	12.7333	0.5434	13.9833	0.4238
21/6/2016	22.2333	1.7802	22.4167	2.0151
27/6/2016	23.7833	1.3926	24.8167	2.2148
1/7/2016	29.45	1.0255	30.8667	1.4159
8/7/2016	25.3167	0.7428	25.4833	1.4013
25/7/2016	26.6	0.0882	28.9	0.1202
27/7/2016	28.6667	1.4121	28.95	1.3979
5/8/2016	26.75	0.4543	28.2333	0.6773
21/8/2016	31.35	2.0742	30.9167	1.1116
22/8/2016	23.7833	1.0398	24.65	0.4899
26/8/2016	17.5667	1.1076	18.5833	0.7796
7/9/2016	28.9667	0.1386	26.9167	0.1814
8/9/2016	21.05	0.6205	22.4833	0.1729
13/9/2016	18.6667	0.3572	20.1833	0.2271
17/9/2016	18.5667	0.8536	19.5667	0.688
19/9/2016	19.4	0.4701	20.45	0.4434
20/9/2016	16.3	0.1567	17.0333	0.073
21/9/2016	18.6667	0.4472	20.1667	0.4725
22/9/2016	17.9	1.1488	18.8333	0.6913
7/10/2016	14.7167	0.3129	15.5333	0.1434
8/10/2016	15.2333	0.6129	15.7833	0.2415
18/10/2016	14.65	0.2797	16.25	0.1265
30/10/2016	8.3	1.0195	8.9	0.8903
16/11/2016	9.7333	0.9924	10.2833	1.4163

Soil moisture_Upper/WFPS%	SE1	Soil moisture_Lower/WFPS%	SE2
26.3209	1.5137	28.492	3.1968
18.4202	1.6149	19.8079	1.8276
31.1106	1.1211	23.6799	1.6482
20.9493	1.973	23.568	1.6331
19.8974	1.9884	16.652	2.6628
31.1106	1.5471	34.4679	1.9863
17.4802	1.2772	22.6951	1.5838
30.4168	1.433	32.3864	3.1902
21.2627	2.1774	23.7694	1.4677
42.9058	1.0344	41.3391	1.6703
39.28	2.1794	38.1609	1.4269
35.5422	2.2284	38.2952	2.6976
47.2927	1.7037	48.2998	1.5135
39.7948	1.4995	40.9139	1.309
27.261	1.3939	30.5735	3.1044
27.0372	2.3127	27.3953	1.7406
9.2437	1.9497	13.0709	1.8497
38.2728	2.0093	43.152	1.8551
29.5215	1.1999	40.8243	1.9812
23.0084	1.4993	31.8716	1.0014
39.1233	2.5111	45.4797	2.2021
39.3919	1.0085	43.5101	1.88
32.3416	1.382	41.5853	1.15
28.5815	1.7121	34.9603	2.6364
36.527	1.3988	40.9139	1.1358
25.2466	0.7257	26.9253	1.3226
24.4633	0.4082	31.4911	1.2728
34.0427	0.8057	41.3839	2.0222
30.4392	1.5903	38.3399	1.1619
31.6254	1.4886	37.0194	1.1225
18.1292	1.2152	22.2922	1.1026
13.4291	3.5773	14.5545	2.5696
25.9628	2.1751	34.647	2.9799
20.6807	3.5286	25.0228	1.3636
30.7302	1.9401	30.2601	2.0942
19.114	2.4255	18.6888	1.9788
33.0355	1.8871	38.2728	1.3627
10.8328	1.5719	10.9223	2.7593
10.1837	2.418	11.0342	1.6027
11.9071	2.344	18.174	1.3856
11.1014	1.2094	16.2492	2.364
39.8843	1.7019	44.9426	1.6088
11.4694	0.8036	14.9063	0.7405
12.3995	0.6928	17.7711	0.971
23.7694	0.6686	31.7821	1.1476
27.3953	1.0113	24.6423	1.6881

22.9637	3.0538	23.2323	1.7308
25.2019	1.7378	26.6343	2.0705
34.0427	1.5132	34.8708	2.8977
27.9772	2.2493	33.4831	1.8048
11.3699	1.2826	12.7576	1.2163
25.2466	1.3743	26.9253	1.3191
26.7462	1.2182	33.2369	1.2349
22.807	2.4295	20.5465	1.5759
34.2217	2.2953	35.7437	1.5829
21.7551	1.4665	19.0693	2.3232
19.114		18.6888	
12.4443	0.0577	13.1381	1.0739
10.1837	0.8311	11.0342	0.8031
26.5224	0.2462	28.8725	2.0415
37.5342	2.2022	40.1081	4.38
21.5313	1.2258	17.5473	1.4599
10.8775	2.2175	11.7728	1.3874
11.4694	2.0942	14.9063	2.952
42.8163	1.647	42.4582	1.3928
24.5976	1.775	24.3514	1.1263
25.4257	1.1184	27.0819	1.3328
27.3953	2.503	24.6423	2.3408
25.2019	2.4712	26.6343	3.4628
34.0427	4.0792	34.8708	1.8306
34.097	3.303	33.0579	1.7918
33.9979	2.1892	34.1546	4.0025
31.6702	2.2693	30.5511	2.7294
43.1744	1.9271	42.8163	3.4297
39.4367	0.9633	39.6829	0.6861
27.7981	3.1288	35.0498	2.968
38.4519	2.2573	42.1225	2.765
26.7014		29.4096	

Rs_Upper/µmol m-2 s-1	SE1	Rs_Lower/µmol m-2 s-1	SE2
1.09	0.0695	3.4275	0.183
0.875	0.2334	2.0825	0.3799
1.675	0.6713	2.725	0.576
1.39	0.1371	2.825	0.1405
2.435	0.066	3.38	0.3927
1.8525	0.1464	3.475	0.2226
1.6175	0.2148	3.3667	0.3774
3.2725	0.1846	4.3825	0.8416
2.6225	1.2745	3.725	0.2105
1.45	0.2514	3.765	0.411
3.415	0.2472	4.9	0.1495
2.805	0.3695	3.745	0.3841
1.79	0.1486	3.35	0.0759
5.225	0.1626	6.375	0.2931
5.055	0.4498	6.55	0.4027
5.1125	0.0635	6.5325	0.0927
1.1375	0	2.4775	0.693
3.125	0.3641	4.52	0.2035
3.3225	0.0794	4.9925	0.3262
2.1933	0.1518	3.71	0.4818
1.55	0.1806	2.9667	0.0492
1.145	0.0472	2.425	0.0458
1.015	0.1013	2.3625	0.0365
1.075	0.079	1.925	0.1085
0.5675	0.1606	1.675	0.1291
0.262	0.0763	0.33	0.0789
0.37	0.1228	0.83	0.1615
0.9183	0.2376	1.725	0.1382
0.87	0.1471	1.43	0.2868
0.7925	0.0988	1.4075	0.3576
1.0975	0.1302	2.1475	0.2738
0.95	0.2295	2.025	0.4714
2.9175	0.2914	4.155	0.4138
2.045	0.0566	3.715	0.0141
2.2575	0.2085	3.7325	0.3203
2.2625	0.1131	3.3	0.2192
2.9725	0.0141	4.2475	0.1273
1.0225	0.0416	2.225	7.07E-03
1.425	0.0424	1.905	0.0283
1.75	0.05	2.97	0.0493
2.0275	0.2313	2.92	0.2996
0.75	0.2765	1.6	0.0678
1.29	0.11	2.125	0.1533
1.33	1.3257	2.205	0.1575
1.53	0.2656	2.35	0.1846
2.7725	0.1678	3.1575	0.067

2.2475	0.452	3.175	0.4588
1.7075	0.1714	3.1	1.0928
1.2375	0.2323	2.8325	0.1658
0.7633	0.2169	1.465	0.065
0.39	0.4639	0.84	0.724
-0.0275	0.2722	0.6333	0.1168
1.065	0.0585	1.5025	0.3057
0.57	0.3604	1.95	0.4957
1.3075	0.5512	2.595	0.5587
2.985	1.1826	3.65	0.3293
2.2625	0.978	2.93	0.2716
1.2675	0.637	2.265	0.1898
1.425	1.6532	1.805	0.484
4.435	1.4119	5.8325	0.289
5.2575	1.4364	6.5225	0.7644
2.24	1.2519	4.595	0.2326
1.57	0.2687	2.3725	0.0919
1.29	0.9649	2.125	0.5709
0.7875	0.7351	1.9725	0.101
3.125	0.3626	5.0875	0.3119
3.5025	0.3606	4.2625	0.997
2.7725	0.2918	3.1575	0.1445
1.7075	0.2642	3.1	0.1112
1.2375	0.0896	2.8325	0.1436
0.955	0.3217	2.435	1.6018
2.885	0.2237	3.85	0.195
2.215	0.2136	3.365	0.306
0.5975	0.1297	1.825	0.5784
1.3675	0.3226	2.75	0.2916
1.15	0.0785	1.91	0.2387
0.56	0.175	1.24	0.8784
0.9	0.1266	1.525	0.3975
