# Supplementary material for: Spatial variations of soil respiration and temperature sensitivity along a steep slope of the semiarid Loess Plateau
Source: PLoS One. 2018 Apr 6;13(4):e0195400. doi: 10.1371/journal.pone.0195400 (PMC5889173; doi:10.1371/journal.pone.0195400)
Supplement: S3 Text — Data for Fig 3. (PDF) [file pone.0195400.s003.pdf]

Bacterial phyla	Abuandance_Upper/%	SE1	Abundance_Lower/%	SE2
Firmicutes	0.9434	0.1643	0.8766	0.053
Verrucomicrobia	1.7793	0.4675	1.9737	0.0883
Bacteroidetes	2.6308	0.2031	2.7147	0.1667
Chloroflexi	3.5677	0.4047	4.2835	0.2272
Nitrospirae	3.6008	0.0796	2.8403	0.0806
Planctomycetes	2.8624	0.8826	3.6767	0.3318
Gemmatimonadetes	10.3509	0.4049	7.7578	0.1607
Actinobacteria	20.0769	2.1749	21.2674	0.6674
Acidobacteria	13.6252	1.7544	18.6025	0.6267
Proteobacteria	36.46	1.83	31.3953	0.2466

Fungal phyla	Abuandance_Upper/%	SE1	Abundance_Lower/%	SE2
Ascomycota	14.5246	4.8316	22.812	7.1643
Zygomycota	4.7184	1.5655	36.4706	8.2984
Basidiomycota	46.8281	6.7038	10.9062	1.348
